# The Role of Sleep in Learning New Meanings for Familiar Words through Stories

**DOI:** 10.5334/joc.282

**Published:** 2023-06-15

**Authors:** Rachael C. Hulme, Jennifer M. Rodd

**Affiliations:** 1 Department of Experimental Psychology, University College London, London, UK

**Keywords:** word learning, sleep, homonyms, story reading, lexical ambiguity, semantic ambiguity

## Abstract

Adults often learn new meanings for familiar words, and in doing so they must integrate information about the newly-acquired meanings with existing knowledge about the prior meanings of the words in their mental lexicon. Numerous studies have confirmed the importance of sleep for learning novel word forms (e.g., “cathedruke”) either with or without associated meanings. By teaching participants new meanings for familiar word forms, this is the first study to focus exclusively on the specific role of sleep on learning word meanings. In two experiments participants were trained on new meanings for familiar words through a naturalistic story reading paradigm to minimize explicit learning strategies. Experiment 1 confirmed the benefit of sleep for recall and recognition of word meanings, with better retention after 12 hours including overnight sleep than 12 hours awake. Experiment 2, which was preregistered, further explored this sleep benefit. Recall performance was best in the condition in which participants slept immediately after exposure and were tested soon after they woke up, compared with three conditions which all included an extended period of wake during which they would encounter their normal language environment. The results are consistent with the view that, at least under these learning conditions, a benefit of sleep arises due to passive protection from linguistic interference while asleep, rather than being due to active consolidation.

Vocabulary knowledge continues to develop throughout life as we learn new words and also new meanings for words that are already familiar to us. Due to technological advancement triggering changes in language, adults must often learn new meanings for words that they already know (e.g., the internet-related meaning of “troll” as a person who posts deliberately antagonizing comments online; Hulme et al., 2019; Rodd, 2020). Adults also learn new meanings for familiar words when learning a new subject or hobby (e.g., in gaming “farming” is repetitive gameplay to gain more items/experience; Eligio & Kaschak, 2021; Rodd et al., 2012, 2016), or when joining a new social/geographical community (e.g., the Scots dialect word “piece” meaning a sandwich).

As adults learn new meanings for familiar words, they must integrate information about the newly-learned meanings with their existing knowledge about the prior meanings of words. With such an abundance of information stored in the mental lexicon, it is a challenge for the learner to acquire these new meanings whilst preserving their knowledge of the pre-existing meanings. The present experiments investigate the potential role of overnight consolidation during sleep in adults’ learning of new meanings for familiar words.

The Complementary Learning Systems (CLS) theory of word learning (Davis & Gaskell, 2009) provides an explanation for how new vocabulary may be integrated with pre-existing knowledge. Davis and Gaskell (2009) combined the CLS model of learning and memory (McClelland et al., 1995) with behavioral and neural findings from studies on spoken word form learning, to develop an account of how adults process and learn new words. They suggest that knowledge about new words is initially encoded into episodic memory in the hippocampus, but only becomes integrated into semantic memory in the neocortex after an active period of offline, sleep-related consolidation. It is only once words have become assimilated into the mental lexicon that they are more rapidly recognized and able to compete with existing similar word forms during word recognition (Tamminen & Gaskell, 2013; Leach & Samuel, 2007). The CLS theory of word learning follows a long tradition of systems consolidation theories proposing an active role for sleep in memory consolidation (Dudai, 1996, 2004; Marr, 1970; McClelland et al., 1995). The CLS model is also closely related to the active systems consolidation (ASC) model (Born & Wilhelm, 2012; Diekelmann & Born, 2010), but differs in terms of the precise mechanisms that are involved in the transfer of memories from the hippocampus to the neocortex.

The CLS model therefore proposes an active role for sleep in the consolidation of novel vocabulary items into semantic memory. In this view, sleep actively protects new vocabulary items from interference by strengthening and stabilizing memory traces. The active account predicts a benefit for words learned immediately prior to a period of sleep because these items are likely to still be available in episodic memory and so can be effectively consolidated during sleep, compared with items learned earlier in the day, which may not be available for effective consolidation. However, an alternative account is that sleep offers a passive benefit to memory: words are better retained when they are encountered immediately before a period of sleep because they are protected against interference from subsequent linguistic input that usually occurs during a period of wake (Jenkins & Dallenbach, 1924). There is a longstanding debate surrounding the nature of the benefit of sleep for memory (for a review see Ellenbogen, Payne, et al., 2006), however more recent research has provided strong evidence for an active role for sleep. For example, manipulating brain oscillations has been shown to enhance memory of word pairs (Ngo et al., 2013), and targeted memory reactivation has been found to facilitate consolidation of picture-location associations (Cairney et al., 2014). However, the importance of passive benefits of sleep on word learning should not be discounted.

Evidence for the CLS theory largely comes from studies of spoken word form learning. For example, Gaskell & Dumay (2003), taught participants novel word forms that were phonological neighbors to existing words (e.g., *cathedruke* for *cathedral*). Participants’ knowledge of these new words was then tested using a recognition test of the new word form, and lexical decision or pause detection tests of the existing words as measures of competition with the new words due to their lexicalization (Takashima et al., 2014), immediately and eight days later. Gaskell and Dumay (2003) found inhibited access for the existing words (due to competition for access from the new word forms) at the delayed test but not immediately after training, suggesting that offline consolidation is required for words to become integrated into the mental lexicon (Tamminen & Gaskell, 2013). Further studies of this nature have replicated these findings (e.g., Davis et al., 2009; Dumay & Gaskell, 2007; Tamminen et al., 2010; Tamminen & Gaskell, 2008). Several of these studies have also specifically shown the importance of sleep for the consolidation process, by dissociating sleep from the simple passage of time (Dumay & Gaskell, 2007), and showing associations between specific components of sleep, such as sleep spindles, and lexical integration (Tamminen et al., 2010). This body of work therefore provides evidence supporting the CLS account of word learning, and suggests that sleep may be critical for lexical consolidation.

However, several more recent studies have provided evidence for the lexicalization of new words without sleep. For example, Kapnoula et al. (2015) trained participants on short non-words that differed from real words on the final phoneme (e.g., *jod* and *job*), and tested for inhibition effects from these novel word forms on the existing words using the visual world eye-tracking paradigm (Tanenhaus et al., 1995). In the experiment they used a phoneme splicing manipulation to amplify competition between the new words and their pre-existing competitors. This competition was measured by examining participants’ eye movements towards a picture representing the existing word which was presenting alongside pictures denoting three unrelated filler words (one with some phonological overlap). They found that the new words were able to compete with the existing words for access immediately after training, without any consolidation (Kapnoula et al., 2015). Kapnoula and McMurray (2016) later provided further evidence that this competition seems to derive from lexicalized representations of the novel words, rather than from episodic memories, in contrast to CLS predictions. In a replication of the previously mentioned *cathedruke* study, Lindsay and Gaskell (2013) also found that words could be integrated immediately, without a period of sleep-based consolidation, when the novel words were repetitively trained using spaced learning alongside exposure to their existing competitor words. Therefore, under certain circumstances immediate integration of novel word forms seems to be possible without consolidation during sleep.

However, studies of learning word forms in isolation are not ecologically valid as word forms are not learned without meanings in everyday life, and this may lead participants to engage in more deliberate memorization than would occur in natural lexical learning. Most new vocabulary is learned incidentally from natural linguistic environments (e.g., conversations, books, TV) in everyday life (Nagy et al., 1985, 1987); however, most studies of vocabulary learning in adults have used rather artificial stimuli, tasks, and learning conditions. One study by (Henderson et al., 2015) examined adults’ and children’s vocabulary learning from naturalistic story reading; they found that novel words only became integrated with existing lexical knowledge after offline consolidation. Importantly, increasing the richness of information about a novel word by adding semantic information has also been shown to differentially engage the complementary memory systems during lexicalization (Takashima et al., 2014), compared to word form learning alone. It is therefore necessary to consider the implications of the CLS account for the learning of novel word meanings. Existing research has combined learning of new word forms with corresponding semantic information, for example by training participants on pronounceable non-words with invented picturable meanings (Clay et al., 2007), meaningful affixes attached to existing words (Tamminen et al., 2012), or low-frequency existing words (van der Ven et al., 2015). Each of these studies found that effects of semantic integration only arose following a period of overnight consolidation, which suggests that information about a word’s meaning also requires time to become integrated into semantic memory (van der Ven et al., 2015), and is consistent with the CLS theory.

Nevertheless, as with word form learning alone, evidence has also been found for the semantic integration of novel words and their meanings without sleep-dependent consolidation, such as in the area of language production (Oppenheim, 2015). Others have suggested that the method of encoding of novel words and their meanings can greatly impact upon subsequent semantic integration (Coutanche & Thompson-Schill, 2014; Himmer et al., 2017). Coutanche and Thompson-Schill (2014) showed that using the fast-mapping learning procedure (whereby participants are forced to infer meaning by process of elimination) enabled immediate lexical integration, while the more traditional explicit encoding procedure produced integration only after consolidation. Offline consolidation may therefore not be a prerequisite for the integration of new knowledge when the learning conditions encourage connections to be formed online between the new information and existing knowledge (Fang et al., 2017; Himmer et al., 2017), as is the case with fast mapping (Coutanche & Thompson-Schill, 2014; Himmer et al., 2017), spaced learning (Lindsay & Gaskell, 2009), and test-enhanced learning (Antony et al., 2017).

However, all of the aforementioned studies of word meaning learning and consolidation combined the acquisition of a new meaning with simultaneous acquisition of a novel word form, which is different to learning a new meaning for an existing word form that already has semantic information attached to it. This is an important distinction, because if both are learned together it is hard to disentangle whether the consolidation effects reflect learning of the form or the meaning, or both. So far only a few studies (Fang et al., 2017; Fang & Perfetti, 2017; Hulme et al., 2019; Hulme & Rodd, 2021; Maciejewski et al., 2020; Rodd et al., 2012) have looked at the effects of making an unambiguous word into an ambiguous one by assigning it a novel invented meaning. These studies did not examine any potential effects of overnight consolidation of the new meanings directly; while Fang and Perfetti (2017) did measure memory immediately after training and one week later, they did not examine the role of sleep and their study cannot disentangle the role of sleep from the passage of time.

The present experiments investigated the impact of sleep on learning new meanings for familiar words acquired incidentally through story reading. Experiment 1 compared participants’ memory of new meanings for familiar words after 12 hours including sleep to 12 hours of wake. Building on this, Experiment 2 compared participants’ memory of new word meanings trained either 24 hours or 12 hours prior to test, with participants tested either in the morning or in the evening to try to tease apart active and passive benefits of overnight sleep.

## Experiment 1

**Figure 1:**
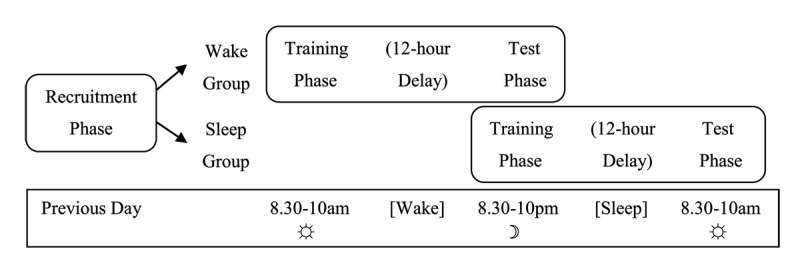
Diagram demonstrating the procedural design for the two groups in Experiment 1.

Experiment 1 examined whether overnight consolidation is beneficial for learning new meanings for familiar words. The experiment used a between-groups design in which participants were trained on new meanings for familiar words incidentally through reading stories (as in Hulme et al., 2019; Hulme & Rodd, 2021). Relatively few studies have previously used such naturalistic, incidental training paradigms to investigate offline consolidation effects on vocabulary learning (Henderson et al., 2015). Participants were trained either in the evening or the morning (see Figure 1). This was then followed by a delay of 12 hours of either sleep or wake, and then by a test session.

Participants’ explicit knowledge of the new meanings was assessed using a cued recall test in which they were required to generate the word’s new meaning in response to its printed form, and a multiple-choice meaning-to-word matching test in which participants were presented with a short definition of each new word meaning and required to select the correct word form from all eight trained word forms. We included both of these explicit memory measures, as productive measures of vocabulary knowledge like cued recall and receptive measures of vocabulary knowledge like multiple-choice recognition can be differentially sensitive depending on the overall level of learning. Both measures are therefore useful together to ensure sufficient sensitivity to detect potential differences regardless of overall performance level. In addition, we included an implicit measure of reaction time (RT) on a semantic relatedness judgement task in which participants made speeded judgements about the pre-existing meaning of the target words. This test was intended to provide a measure of semantic integration by measuring the extent to which learning a new meaning for a familiar word form like “foam” would impair participants’ ability access this word’s familiar meaning and judge whether it was related to the probe word (e.g., *foam-soap*/*foam-belt*) (Fang et al., 2017; Fang & Perfetti, 2017; Maciejewski et al., 2020; Rodd et al., 2012). This task was designed to be a semantic analogue of tasks such as pause detection, which has been used in word-form learning studies to measure the impact of newly acquired knowledge about a novel word form (e.g., “cathedruke”) on online processing of a familiar word form (“cathedral”) (Gaskell & Dumay, 2003; Lindsay & Gaskell, 2013). The logic underlying this task fits within the framework of a distributed connectionist account of ambiguous word processing (Rodd, 2022; Rodd et al., 2002, 2004), whereby recognition of words with multiple unrelated meanings is delayed by the competition between the words’ mutually incompatible semantic representations (Rodd et al., 2012).

Based on previous studies of word *form* learning (Dumay et al., 2005; Henderson et al., 2015; Takashima et al., 2014; Tamminen et al., 2010) we predicted that participants who had slept between training and test would have better explicit memory of the new word *meanings* than those who had not slept. In addition, we predicted that participants who had slept would be slower to make a semantic relatedness decision to the trained words, due to increased competition arising between the new and old meanings. Any such inhibition effect would be taken as evidence that the new meanings had not only been better retained after sleep, but were sufficiently well integrated into the lexicon to compete during lexical access.

### Method

#### Participants

We aimed to recruit 80 participants for Experiment 1 (40 participants per group, ten participants per version) in which participants were trained on eight items in a single training session. The sample size was established in consideration of previous word learning studies that have used the same or similar items (Hulme et al., 2019; Hulme & Rodd, 2021; Maciejewski et al., 2020; Rodd et al., 2012).

Eighty-four participants were included in the experiment (age: *M* = 31.4 years, *SD* = 8.6; 39 female). We over-recruited by four participants when assigning participants to the experiment versions and kept these participants. Participants were recruited through Prolific (www.prolific.co) using pre-screening criteria. Participants were eligible to take part if they were a current UK resident, monolingual native speaker of British English, and had no diagnosis of reading or language impairments. None of the participants had been diagnosed with a sleep disorder or were taking medication that could affect their sleep. They gave their informed consent before taking part and were paid £8 for their participation upon completion of all sessions.

An additional 39 participants began the experiment but dropped out before completing all sessions (12 from the wake group, 27 from the sleep group), and 17 participants failed to complete all sessions due to a technical issue; these participants were excluded. Thirteen further participants were excluded for having a sleep disorder or taking medication that could disrupt their sleep. A further 15 participants were excluded for getting more than one of the multiple-choice comprehension questions wrong when reading either of the stories. Four additional participants were excluded for being outliers in their mean reading speed (faster than 624.0 words per minute, two *SD* above the mean). Finally, 11 participants were excluded for misunderstanding the instructions of the cued recall test (e.g., providing a definition for the existing rather than the new meaning of a word), and one participant was excluded for low accuracy on the semantic relatedness judgement task (less than 87.8%, three *SD* below the mean). Excluded participants were replaced during recruitment.

#### Materials

##### Novel word meanings and short stories

The stimuli were 16 previously unambiguous words that were assigned new invented meanings, taken from Hulme et al. (2019). The novel meanings (describing fictional innovations, discoveries, and inventions) were semantically unrelated to the real existing meanings of the words. Single sentence definitions were initially constructed (matched for length: *M* = 32.9 words, *SD* = 3.7) to describe the new meanings for each of the words. These single sentence definitions were not presented to participants, but were provided to the story authors to incorporate the novel words into story narratives. For example, the definition sentence for “foam” was: “A foam is a safe that is incorporated into a piece of furniture with a wooden panel concealing the key lock, and each is individually handcrafted so that no intruders are able to recognize the chief use of the furniture.” (see Table S1 for the list of words and their definitions: https://osf.io/hykq9). The words with new meanings were incorporated into four separate short stories (2307–2446 words in length), taken from Hulme et al. (2019) (see Supplementary Materials: https://osf.io/hykq9). The stories were written for an adult audience by a professional children’s author (Story 1: Pink Candy Dream), and an unpublished author (Story 2: Prisons, Story 3: Reflections upon a Tribe, and Story 4: The Island and Elsewhere). Four of the words and their new meanings were included in each story, with eight occurrences of each item throughout the narrative. The first occurrence of an item gave sufficient information to allow participants to derive the new meaning from the context, for example, “‘Yes,’ I murmured, breathing again. ‘I knew it! It’s a foam.’ The ornate chaise longue was no ordinary piece of furniture but concealed a built-in safe with an intricate key-operated locking system.” The amount of information about each new meaning in subsequent occurrences varied naturally according to the narratives.

Additionally, paraphrased definition sentences that were used in the cued recall of word forms test in Hulme et al.’s (2019) study were used in the multiple-choice meaning-to-word matching test in this experiment (see Table S2: https://osf.io/hykq9). These short, paraphrased definition sentences were presented to participants in the multiple-choice meaning-to-word matching test and participants were asked to select the appropriate word for each definition.

##### Semantic relatedness judgement task

Each of the 16 stimulus words was paired with one probe word that was semantically related to the existing meaning of the word (e.g., *foam-soap*) and one that was semantically unrelated (e.g., *foam-belt*; see Table S3 for the full list of target words and probes: https://osf.io/hykq9). For each participant, the eight items that they were not trained on (in the two stories they did not read) served as untrained control items (with the items in each training condition counterbalanced across participants). The majority of the semantically related probes (*n* = 10) and unrelated probes (*n* = 12) were selected from those used by Maciejewski et al. (2020) paired with the same target words in their semantic relatedness judgement task. The remaining semantically related probes (*n* = 6) were selected from the Edinburgh Association Thesaurus (EAT; Kiss, Armstrong, Milroy, & Piper, 1973), and the remaining unrelated probes (*n* = 4) were selected from the remaining set of probe words from Maciejewski et al. (2020) that had been used with other target words. The degree of semantic relatedness between each target word and its corresponding related and unrelated probes was determined using Latent Semantic Analysis (LSA; Landauer, Foltz, & Laham, 1998). The mean LSA score comparing semantically related probes and targets was 0.4 (*SD* = 0.2), and for semantically unrelated probes and targets it was 0.06 (*SD* = 0.1). All of the probe words were nouns, and the related and unrelated probes were matched as closely as possible to both the target words and to each other in terms of frequency and word length, and were also similar in terms of number of senses and number of semantic associates (see Table S4 for the properties of the probe words: https://osf.io/hykq9). The probe words were not semantically related to the novel meanings of the words, and none of the probe words appeared in any of the stories.

Additionally, 16 fillers were selected from the control words used by Maciejewski et al. (2020) that were also matched to the stimuli by frequency and word length. Half of these fillers (selected at random) were paired with two semantically related probes, and the other half were paired with two semantically unrelated probes. This was to prevent a predictable pattern whereby each target word seen would appear once with a related probe followed by an unrelated probe, or vice versa. The probes for the fillers were selected as above. An extra eight unmatched fillers served as a practice block before the start of the main experimental task. These fillers had the same distribution as the trials in the main experiment: half were paired with both a related and unrelated probe, and half were paired with either two related or two unrelated probes. Another eight extra unmatched fillers, with the same distribution of target-probe pairings were selected to appear at the beginning of the experimental task blocks to accustom participants to the speed and rhythm of the task.

#### Design

Sleep was manipulated between-participants (wake group vs. sleep group) and within-items. Training condition in the semantic relatedness judgement task (trained items vs. untrained items) was manipulated within-participants and within-items. Each participant was trained on only half the total number of stimuli (eight items) through two of the four stories (Stories 1 and 4, or 2 and 3), as this was deemed to be a feasible number of new meanings to learn in a single session. Furthermore, to ensure that each new word meaning appeared roughly an equal number of times in each sleep condition, and that the order of the two blocks in the semantic relatedness judgement task was counterbalanced across participants (to minimize any order effects), there were eight versions of the experiment. Participants were pseudorandomly assigned to one of the eight versions of the experiment, there were 10–11 participants per version, and half of the participants in each the wake group and the sleep group (42 participants per group). The dependent measures were accuracy in cued recall of meanings and the multiple-choice test, and RT and accuracy in semantic relatedness judgement task.

#### Procedure

The experiment was run online using Qualtrics (Qualtrics, 2015) with the Qualtrics Reaction Time Engine (QRTE; Barnhoorn et al., 2015). Figure 1 shows a schematic of the experiment schedule.

##### Recruitment phase

Participants provided demographic details and confirmed their availability to take part in sessions at all of the possible times (although they would only be required to complete sessions at some of those times). Participants were then pseudorandomly and evenly assigned by the experimental software to one of the eight versions of the experiment, which determined whether they were in the wake group or the sleep group. They were then given the times for their two subsequent sessions starting the following day at either 8.30–10am and 8.30–10pm for the wake group, or 8.30–10pm and 8.30–10am the following morning for the sleep group. Participants were not told that the purpose of the experiment was to learn new word meanings, and were not aware that their memory would be tested. Instead they were told that the experiment investigated reading ability and comprehension at different times of day.

##### Training phase

Participants each read two stories (stories 1 and 4, or 2 and 3). The procedure for reading the stories was the same as described in Hulme et al. (2019). Each story was displayed on-screen across five pages of approximately equal length. After each page, a four-alternative multiple-choice comprehension question on a separate screen asked about details of the story’s plot from the preceding page (without probing details of the novel word meanings). Participants were instructed to read the story carefully, without skim-reading, and to answer the multiple-choice comprehension questions; participants were not able to re-read previous pages. After completing the first story, participants were given a brief break of 20 seconds before they could continue to the second story.

##### Testing phase

At the start of the testing phase participants rated their alertness on the Stanford Sleepiness Scale (SSS; Hoddes et al., 1973).

###### Semantic relatedness judgement

The first test was the semantic relatedness judgement task. Participants were presented with the stimuli and filler words one at a time on the screen. Each trial began with a 500 ms fixation cross, followed by the target word for 500 ms, then a second 500 ms fixation cross, and finally the probe word was presented until a response was given. Participants’ task was to decide whether the target and probe word were semantically related (e.g., *foam-soap*) or not (e.g., *foam-belt*). Participants were not told which meaning of the target word they should attend to, and were instructed to respond as quickly and accurately as possible. They indicated their choice with a “yes” response (“j” key), or a “no” response (“f” key).

Participants first completed a practice block of 16 trials. Following each practice trial, a feedback screen informed them whether their response had been correct or incorrect, and for responses slower than 2000 ms, a feedback message told participants that their response was too slow and they should respond more quickly. Participants then proceeded to the main experimental task. Trials were split into two blocks of 40 trials each, which each began with eight fillers, followed by 16 experimental trials intermixed with 16 matched filler trials. Each target word (stimulus or filler) appeared once in each of the two blocks, paired with one of its two probe words in the first block, and the other in the second block. For the stimulus items, half of the targets (selected at random) appeared with their semantically related probe in Block 1 and then with their semantically unrelated probe in Block 2; and vice versa for the other half. The order of the blocks was counterbalanced across participants, and the order of trials within each block (and the fillers at the beginning of each block) was randomised for each participant. The same feedback message was displayed when participants responded slower than 2000 ms; there was no accuracy feedback in the main task. There was a break of at least ten seconds between the two blocks.

###### Cued recall of meanings

Participants were then given a cued recall test for the newly learned word meanings. They were presented one at a time with the eight words they had been trained on and instructed to type the new meaning for the word into a text box. They were asked to give as much detail as they could and to try to answer in full sentences even if they were unsure of their answer. If they could not remember anything about the new meaning, they were instructed to type “don’t know”. The order of presentation of the word cues was randomized for each participant.

###### Multiple-choice meaning-to-word matching

Finally, participants completed the multiple-choice meaning-to-word matching test. Participants were presented one at a time with short sentences giving definitions of the each of the new word meanings that they had been trained on. For each novel meaning participants were asked to select the word that matched the definition from a list of all eight of the words they had been trained on. The order of these eight words was randomized for each test item, and the order of the new meaning definitions was randomized for each participant.

### Results

#### Stanford Sleepiness Scale

Results for the SSS (Hoddes et al., 1973) measured at the test session were analyzed using a Wilcoxon-Mann-Whitney test. There was no significant difference in SSS score between the wake group (*Mdn* = 3) who were tested in the evening, and the sleep group (*Mdn* = 3) who were tested in the morning [W = 798, *p* = .437].

#### Analysis procedure

Responses for the cued recall test were coded by the experimenter blind to condition as “1” for correctly recalled items or “0” for incorrect. Responses were leniently coded as correct if at least one correct feature of a new meaning was recalled, and any ambiguous or partially correct responses were resolved on a case-by-case basis.^1^ Responses from the multiple-choice test were coded as “1” if the correct word had been selected, or “0” for incorrect.

Data from the semantic relatedness judgement task were pre-processed prior to analysis. Accuracy was very high overall for all items in the semantic relatedness judgement task.^2^ For analysis of the RT data incorrect trials were removed (2.6% of trials), and RTs faster than 300 ms or slower than 2500 ms were trimmed from the data (0.2% of remaining trials). The RT analysis was only for correct related trials, for which participants had correctly responded that the target and probe words were semantically related. The accuracy analysis was also only for related trials, with RTs faster than 300 ms or slower than 2500 ms trimmed from the data.

Data from the three test tasks were analyzed separately using linear mixed effects (LME) models using the *lme4* package (version 1.1-12; Bates et al., 2015) and R (version 3.3.1; R Core Team, 2017). The binary accuracy data from the cued recall and multiple-choice measures were analyzed using logistic LME models. The contrast for the fixed effect of group was defined using deviation coding (sleep group: 0.5, wake group: –0.5). These two models included by-participant and by-items random intercepts, and a by-items random slope for group. The random effects structure was determined by identifying the maximal random effects structure justified by the design (Barr et al., 2013).

The RT and accuracy data from the semantic relatedness judgement task were analyzed using an LME model and logistic LME model respectively. Contrasts for the fixed effects were defined using deviation coding for group (sleep group: 0.5, wake group: –0.5), training condition (trained items: 0.5, untrained items: –0.5), and block (first block: –0.5, second block: 0.5),^3^ with the interactions coded by multiplying the contrasts for the appropriate factors. Both models contained fixed effects for group, training condition, and block, as well as all of the two-way interactions, and the three-way interaction. The maximal random effects structure was first fitted for both models, consisting of by-participant and by-items random intercepts, by-items random slopes for group, training condition, block, all two-way interactions, and the three-way interaction, and by-participant random slopes for training condition, block, and the interaction. This model converged and was used as the final model for the RT analysis; the model for analysis of the accuracy data was simplified by removing the correlations between the by-participant and by-item random slopes and random intercepts (as recommended by Barr et al., 2013). The assumptions of homoscedasticity and normality were violated in the raw RT data, so the data were log_10_- and inverse-transformed (invRT = 1000/rawRT) and compared with the raw RT data. The inverse-transformed RTs met the assumptions of homoscedasticity and normality most closely and were therefore used for the analysis. Significance of the fixed effects and interactions was assessed using likelihood ratio tests comparing the full model to models with only each fixed factor/interaction of interest removed in turn. Significance for follow-up analyses was assessed using the same method. Data and analysis scripts for this experiment are available via the Open Science Framework (OSF; https://osf.io/m3pj6).

#### Cued recall of meanings

**Figure 2:**
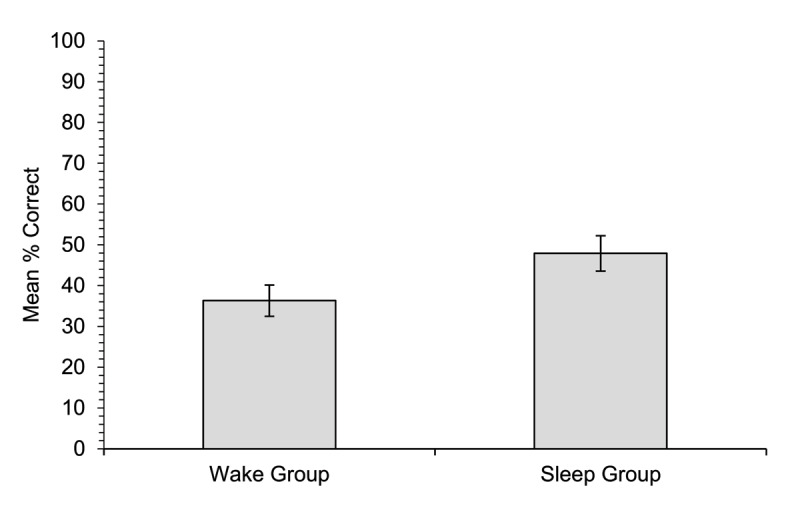
Experiment 1. Mean percentage of correct responses on the cued recall test (meanings correctly recalled for the appropriate word) for participants in each of the two groups. Error bars show standard errors for the means.

Accuracy on the cued recall test (Figure 2) was low overall: participants in both groups could generate the meaning for less than 50% of the words. The sleep group correctly recalled significantly more of the novel word meanings (47.9%) than the wake group (36.3%), [*χ*^2^(1) = 4.13, *p* = .042].

#### Multiple-choice meaning-to-word matching

**Figure 3:**
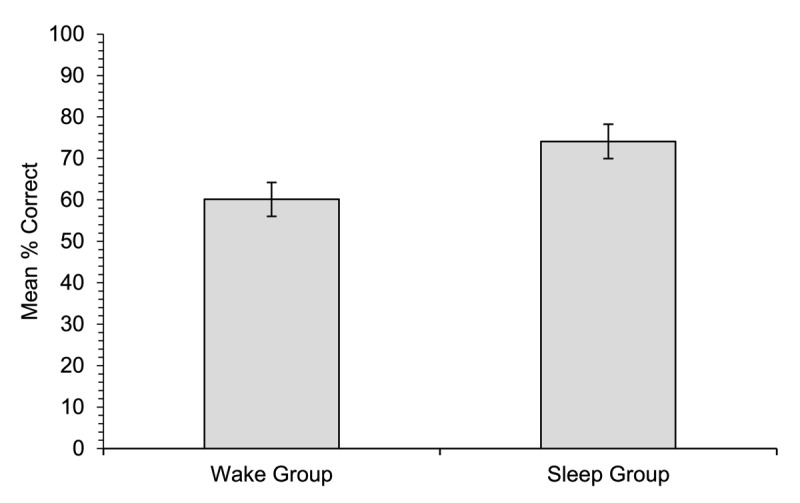
Experiment 1. Mean percentage of correct responses on the multiple-choice meaning-to-word matching test (words correctly matched with the appropriate meaning) for participants in each of the two groups. Error bars show standard errors for the means.

Data for the multiple-choice test (Figure 3) showed higher overall accuracy than for cued recall, but it was not near ceiling. The pattern of the data was the same as for the cued recall test: accuracy was significantly higher for the sleep group (74.1%) than for the wake group (60.1%), [*χ*^2^(1) = 7.01, *p* = .008].

#### Semantic relatedness judgement

**Figure 4:**
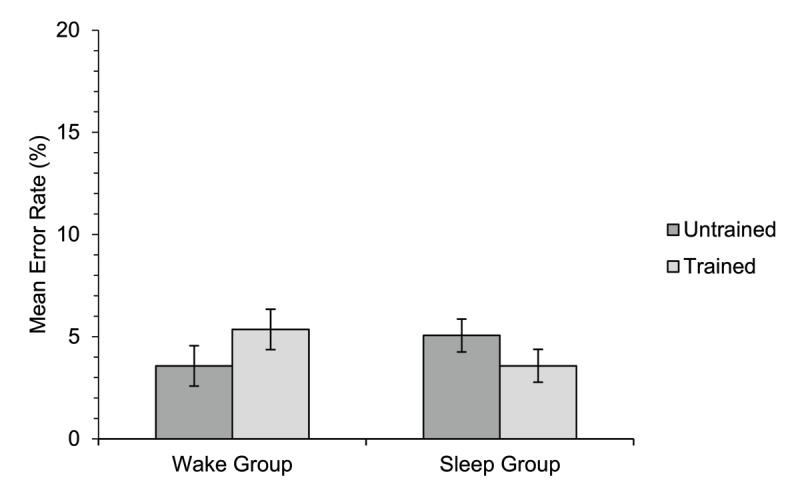
Experiment 1. Mean percentage error rate on the semantic relatedness judgement task for participants in each of the two groups for untrained and trained items. The data shown are related trials only (trials in which the target and probe were semantically related). Error bars show standard errors for the means, corrected for the within-participants factor of training condition (Cousineau, 2005).

Mean percentage error rates for the semantic relatedness judgement task are illustrated in Figure 4; accuracy was very high overall and error rates differed little between the different conditions. There was no significant overall difference in accuracy between the two groups [*χ*^2^(1) = 0.03, *p* = .868], and no significant overall difference between accuracy for trained and untrained items [*χ*^2^(1) = 0.001, *p* = .972]. The interaction between group and training condition was non-significant [*χ*^2^(1) = 0.80, *p* = .370]. There was also no significant effect of block [*χ*^2^(1) = 0.69, *p* = .408], interaction between group and block [*χ*^2^(1) = 0.83, *p* = .361], interaction between training condition and block [*χ*^2^(1) = 0.08, *p* = .778], or three-way interaction [*χ*^2^(1) = 2.69, *p* = .101].

**Figure 5:**
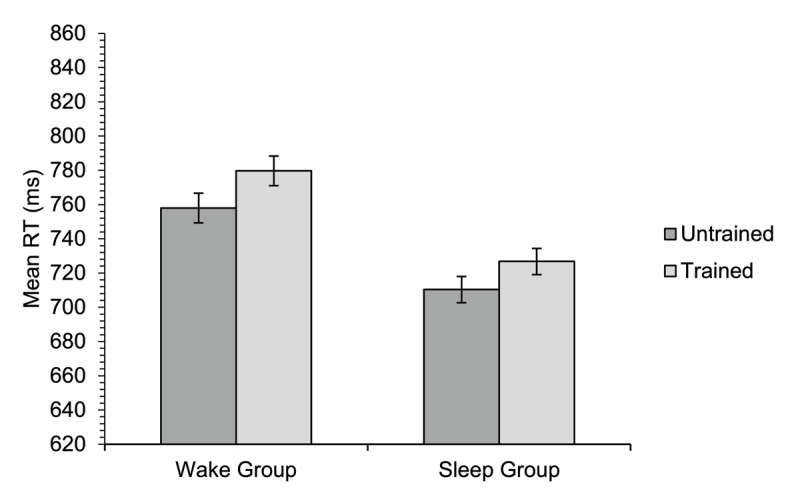
Experiment 1. Mean reaction time on the semantic relatedness judgement task for participants in each of the two groups for untrained and trained items. The data shown are correct related trials only (trials to which the participants correctly responded ‘yes’ that the target and probe were semantically related). Error bars show standard errors for the means, corrected for the within-participants factor of training condition (Cousineau, 2005).

The RT data from the semantic relatedness judgement task are shown in Figure 5. There was a significant main effect of group [*χ*^2^(1) = 3.90, *p* = .048], whereby the sleep group, who were tested in the morning, were faster overall than the wake group, who were tested in the evening. Although there was a trend in the data for participants in both groups responding slightly slower to trained items than untrained items, the main effect of training was non-significant [*χ*^2^(1) = 1.60, *p* = .205]. Furthermore, the predicted interaction between group and training condition was not significant [*χ*^2^(1) = 0.09, *p* = .769]. There was also no significant effect of block [*χ*^2^(1) = 0.06, *p* = .810], interaction between group and block [*χ*^2^(1) = 0.92, *p* = .338], interaction between training condition and block [*χ*^2^(1) = 0.06, *p* = .809], or three-way interaction [*χ*^2^(1) = 0.23, *p* = .632].

To obtain a full impression of the data from the semantic relatedness judgement task, we carried out additional exploratory analyses with a subset of trained items for which participants had correctly recalled the item in the cued recall test (similar to Rodd et al., 2012; Experiment 3). Results were largely similar to the main analyses (see Supplementary Results: https://osf.io/p3t49).

### Discussion

Experiment 1 investigated how the contribution from a delay including sleep compares to a delay without sleep for learning new word meanings. Participants were trained on new meanings for familiar words incidentally through story reading. The sleep group were trained in the evening and tested the next morning after a 12-hour delay. The wake group were trained in the morning and tested that evening, also after a 12-hour delay. Results for the two explicit memory measures showed that participants in the sleep group remembered significantly more of the new word meanings than those in the wake group. Mean accuracy in cued recall of new meanings was 11.6% higher for the sleep group than the wake group, and accuracy on the multiple-choice test was 14.0% higher for the sleep group than the wake group.

These findings for word meanings are broadly consistent with other similar studies of word-form learning that show better retention after 12 hours including sleep than after 12 hours of wake (Dumay & Gaskell, 2007; Tamminen et al., 2010). For example, Tamminen et al. (2010) found significantly improved recall of new word forms for a group tested after 12 hours including sleep, but no improvement for a group tested after 12 hours of wake.

In contrast to these clear effects in the explicit measures of word meaning learning, the semantic relatedness judgement task showed no significant effect of training and no significant interaction between training and sleep group. It is unclear whether these null effects reflect the true absence of integration/consolidation of these new word meanings into the lexicon, or a lack of task sensitivity in the current paradigm to detect such consolidation effects.

A possible limitation of Experiment 1 lies in participant drop-out from the experiment. Although participants were randomly assigned to the sleep and wake groups during the recruitment phase of the experiment, there was some selective attrition. More participants dropped out from the sleep group, for whom the two experimental sessions were on two separate days, than the wake group for whom both experimental sessions were on the same day. This is potentially problematic as selective attrition could introduce confounds (e.g., participant motivation) of the experimental manipulation (Zhou & Fishbach, 2016). The pattern of results for Experiment 1 could potentially be partially explained by non-random participant drop-out from the sleep group if, for example, the higher attrition rate in the two-day version of the task selectively excluded participants who were less motivated to learn.

In summary, participants had better explicit memory of new meanings for familiar words after 12 hours that included a period of overnight sleep, as compared with participants tested after a 12-hour period of wake. However, in the absence of significant effects on the implicit measure of consolidation of the new meanings, it is unclear whether this sleep benefit was due to active sleep-related consolidation or due to passive protection from interference from the linguistic input that participants in the wake group will have encountered during their day. Furthermore, the sleep effect seen in recall and recognition of new word meanings may be an effect of time of day, for example due to general cognitive enhancement improving recall function (Schmidt et al., 2007), as time of day was confounded with sleep group at both encoding and test. It is possible that due to circadian differences participants may have learned better in the evening, or remembered better in the morning. However, there was no significant difference in participants’ SSS ratings (Hoddes et al., 1973) between the sleep group and the wake group at the test session.

## Experiment 2

**Figure 6:**
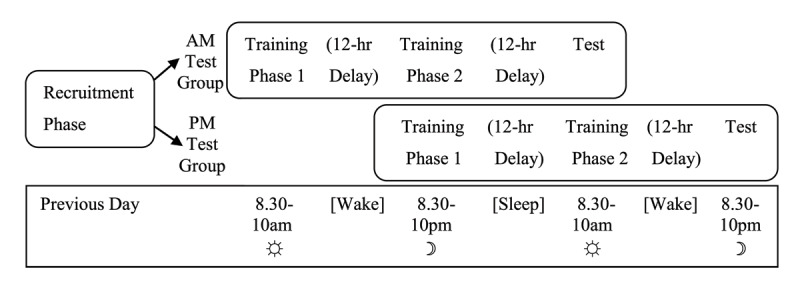
Diagram demonstrating the procedural design for the two groups in Experiment 2.

The aim of Experiment 2 was to further investigate the sleep benefit seen in Experiment 1 using a preregistered mixed “12:12” design similar to that used by Dumay and Gaskell (2007). Participants were divided into two groups who began the experiment either in the morning or the evening. Both groups were immediately trained on half the target words and then returned after 12 hours for training on the other half of the target words. After a second 12-hour delay all participants were tested on all items (see Figure 6). The two groups therefore had the same lengths of time delay between the two training sessions and the test (24 hours and 12 hours) and the same amounts of time spent asleep and awake, with the only difference being when the period of sleep occurred in relation to the test. This design allowed for exploration of the effects of sleep without repeated testing of newly learned information (Hulme & Rodd, 2021).

Experiment 2 therefore included the two 12-hour delay conditions from Experiment 1. For these conditions we expected to replicate the sleep benefit seen in Experiment 1, such that the new meanings should be better remembered by the AM-test group who experienced overnight sleep during the 12-hour delay period, compared with the PM-test group who remained awake throughout the delay. Experiment 2 also included two additional conditions in which items are tested 24 hours after learning. Critically, for the PM-test group these items were learned immediately before overnight sleep and then tested 24 hours later such that participants had the opportunity for active sleep-related consolidation that would protect them from forgetting during their subsequent day awake. In contrast, for the AM-test group learning was immediately followed by a period of wake, such that participants would have already (partially) forgotten much of what they learned during the 12 hours that they spent awake after training (as shown in Experiment 1) before they had the opportunity to consolidate this knowledge overnight.

Therefore, if the sleep benefit observed in Experiment 1 was driven by active sleep-related consolidation (Davis & Gaskell, 2009) we should observe an interaction between training condition and group: in the 12-hour delay condition performance should be better for the AM-test group, whereas in the 24-hour delay condition performance should be better for the PM-test group. This account also predicts a similar pattern of results in the semantic relatedness judgement task: competition between the new and old meanings of the words should *only* arise for items learned immediately before overnight sleep, and not for items trained before a 12-hour period of wake.

In contrast, the passive account of the benefit of sleep (Ellenbogen, Payne, et al., 2006) predicts a somewhat different pattern of results. Specifically, this account predicts that memory performance should be best for participants who learned the new word meanings in the evening and were tested the following morning (AM-test group: 12-hour delay), because this is the only experimental condition that does *not* include an extended period of daytime linguistic experience between training and test. Therefore, this condition minimizes the amount of potentially interfering linguistic information that participants encounter between training and test. In contrast, the other three experimental conditions all contain a single period of daytime awake, which is likely to reduce retention of the newly learned word meanings. This account is somewhat underspecified, and it is currently unclear whether these three conditions might also differ from each other due to differences in exactly when this interfering linguistic information is encountered relative to learning and test. Thus although this account also predicts a group by training condition interaction, it predicts that this interaction should take a somewhat different form, such that the two groups should differ in their performance in the 12-hour delay condition, but that this difference should not be present in the 24-hour delay condition. Furthermore, the passive account of the role of sleep is also underspecified as to whether we would expect to observe competition effects between new and old meanings on the semantic relatedness task as it is unclear whether any of these conditions would result in learning that was sufficiently strong to drive competition effects (Rodd et al., 2012). Finally, if the results of Experiment 1 are due to time-of-day effects, then the results of this experiment should show a straightforward pattern whereby the strength of learning depends solely on the time at which participants were trained or tested. This experiment was preregistered through the Open Science Framework: https://osf.io/uvgp4 (Hulme & Rodd, 2017, August 9). Where applicable any deviations from the preregistration have been noted in the Method and Results sections.

### Method

#### Participants

We aimed to recruit eighty participants for Experiment 2 in which participants were trained on 16 items (eight per training session) in one of two groups, with eight experiment versions (ten participants per version). The sample size was established in consideration of Experiment 1 and other previous word learning studies that have used the same or similar items (Hulme et al., 2019; Hulme & Rodd, 2021; Maciejewski et al., 2020; Rodd et al., 2012).

Eighty-four participants were included in the experiment (age: *M* = 34.0 years, *SD* = 7.1, range = 20–48; 69 female); we over-recruited by four participants when assigning participants to the experiment versions and kept these participants. Participants were recruited in the same way as for Experiment 1. They gave their informed consent before taking part and were paid £10 for their participation upon completion of all sessions.

An additional 42 participants began the experiment but dropped out before completing all sessions (23 from the AM-test group, 19 from the PM-test group) and were excluded. Four additional participants were excluded due to a technical error or attempting to complete a session more than once. A further 25 participants were excluded for getting more than one of the multiple-choice comprehension questions wrong when reading any one of the stories. Five participants were excluded for being outliers in their mean reading speed (faster than 657.2 words per minute, two *SD* above the mean). Finally, one participant was excluded for low accuracy on the semantic relatedness judgement task (less than 75.4%, three *SD* below the mean). Excluded participants were replaced during recruitment.

#### Materials

##### Novel word meanings and short stories

The stimulus words, short stories, and definition sentences for the multiple-choice test were identical to those in Experiment 1.

##### Stimuli for semantic relatedness judgement task

The stimuli for the semantic relatedness judgement task were the same as those in Experiment 1, with an additional set of matched control words (see Table S3 for the full list: https://osf.io/hykq9). The experimental trials consisted of the 16 trained target words and their probes from Experiment 1, and eight matched control words that were also paired with both a related and unrelated probe (see Table S5 for the properties of the probe words: https://osf.io/hykq9). There were also 24 fillers, paired with either two semantically related or two semantically unrelated probes, and eight additional fillers each paired with two probes (with the same distribution of probe types as the experimental and control trials) to serve as buffer trials at the start of each experimental block. Finally, another eight fillers were selected and each paired with two probes (with the same distribution of probe types) to serve as practice trials before the start of the experimental blocks.

#### Design

The experiment had a mixed design: group (AM-test group vs. PM-test group) was manipulated between-participants and within-items, training condition (12-hour delay vs. 24-hour delay) was within-participants and within-items (with an additional level of training condition in the semantic relatedness judgement task: untrained, which was within-participants and between-items). We created eight versions of the experiment to ensure that items were trained an even number of times in each group, and that the eight items (Stories 1 and 4, or 2 and 3) trained in each session and the order of the two blocks in the semantic relatedness judgement task were counterbalanced across participants. Participants were pseudorandomly assigned to one of the eight versions in the recruitment phase of the experiment (10–12 participants per version; AM-test group: *N* = 43, PM-test group: *N* = 41).

#### Procedure

The experiment was run online using Qualtrics (Qualtrics, 2015) for the recruitment and training phases, and Gorilla Experiment Builder (www.gorilla.sc; Anwyl-Irvine et al., 2020) for the testing phase. Figure 6 shows a schematic of the experiment schedule.

##### Recruitment phase

As for Experiment 1, the study began with a recruitment phase in which participants provided demographics details and confirmed their availability to take part in sessions at all of the possible times (although they would only be required to complete sessions at some of those times). The participants were then pseudorandomly and evenly assigned by the experimental software to one of the eight versions of the experiment, which determined whether they in the AM-test group or the PM-test group. Participants were given the times for their three subsequent sessions beginning the following day, for the AM-test group these were: 8.30–10am, 8.30–10pm, and 8.30–10am the following day; for the PM-test group these were: 8.30–10pm, and 8.30–10am and 8.30–10pm the following day (see Figure 6). Participants were not informed that the purpose of the study was to examine learning of new word meanings, and were not aware that their memory would be tested. Instead they were told that the experiment investigated reading ability and comprehension of texts at different times of day.

##### Training phase

The training phase consisted of two separate sessions spaced 12 hours apart. At the beginning of each of the two training sessions and the test session participants were asked to rate their alertness on the Stanford Sleepiness Scale (Hoddes et al., 1973). During each training session, participants read two of the short stories (either Stories 1 and 4, or 2 and 3). The procedure for reading the stories and answering the simple multiple-choice comprehension questions was the same as for Experiment 1. In each session, after completing the first story participants answered some questions about their enjoyment and clarity of the story they had just read and their reading habits (taking approximately 30 seconds in total) before they could begin reading the second story. After reading the second story participants were asked the same questions about their enjoyment and the clarity of the second story. The purpose of these questions was to maintain the impression that the purpose of the experiment was to investigate reading ability and comprehension at different times of day.

##### Testing phase

###### Semantic relatedness judgement

The semantic relatedness judgement task from Experiment 1 was adapted slightly to shorten trial duration. Recent studies that have used this task to explore semantic integration effects have used shorter durations for presentation of the target word and inter-stimulus interval (Gilbert et al., 2018; Maciejewski et al., 2020). It was possible that the relatively long delay between the initial onset of the target word and the onset of the probe word reduced the sensitivity of this task in Experiment 1 as disambiguation of the target word may have been fully resolved before the presentation of the probe. We therefore shortened the timing of trial structure to match that used by Maciejewski et al. (2020).

Each trial began with a 500 ms fixation cross, followed by a 100 ms blank screen, the target word then appeared onscreen for 200 ms, followed by another 50 ms blank screen, then the probe word was presented until a response was given (with a time-out after 2000 ms). If a response was not given until after the probe had been onscreen for 1500 ms, then a message was displayed to tell the participant that their response was too slow and that they should respond more quickly.

Trials were split into two blocks of 56 trials each. Each block began with eight fillers, followed by 16 trials where the target was a trained word, intermixed with eight trials with untrained control target words, and 24 matched filler trials. Each target word (stimulus, control, or filler) appeared once in each of the two blocks, paired with one of its two probe words in the first block, and the other in the second block. Half of the stimuli/control words (selected at random) appeared with their semantically related probe in Block 1 and then with their semantically unrelated probe in Block 2; and vice versa for the other half. The order of the blocks was counterbalanced across participants. The order of trials within each block (and the fillers at the beginning of each block) was randomized for each participant. There was a brief break of around ten seconds between blocks.

###### Cued recall of meanings

Participants then completed the cued recall test for all 16 new word meanings that they had been trained on. The procedure was the same as for Experiment 1.

###### Multiple-choice meaning-to-word matching

The procedure for the multiple-choice meaning-to-word matching test was also the same as for Experiment 1, with participants tested on the 16 novel word meanings that they had been trained on. For each novel meaning participants were asked to select the word that matched the definition from a list of the eight stimulus words they had been trained on within the same training session.

### Results

#### Stanford Sleepiness Scale

Results for the SSS (Hoddes et al., 1973) measure taken at the beginning of each session were analyzed using three Wilcoxon-Mann-Whitney tests. The SSS scores at the first training session did not differ between the AM-test group who were trained in the morning (*Mdn* = 3) and the PM-test group who were trained in the evening (*Mdn* = 3), W = 728.5, *p* = .150. The SSS scores for the second training session also did not differ between the AM-test group who were trained in the evening (*Mdn* = 3) and the PM-test group who were trained in the morning (*Mdn* = 2), W = 1038.5, *p* = .149. Finally, SSS scores at the test session did not differ between the AM-test group who were tested in the morning (*Mdn* = 3) and the PM-test group who were tested in the evening (*Mdn* = 3), W = 895.5, *p* = .746.

#### Analysis procedure

Responses for the cued recall and multiple-choice measures were coded as “1” for correct or “0” for incorrect in the same way as for Experiment 1. Data from the semantic relatedness judgement task were pre-processed as for Experiment 1 prior to analysis. For the RT analysis incorrect trials were removed (5.5% of trials), and RTs faster than 300 ms or slower than 2500 ms were trimmed from the data (0.1% of remaining experimental trials). The RT analysis was only of correct, related trials. The accuracy analysis was also only for related trials, with RTs faster than 300 ms or slower than 2500 ms trimmed from the data.

Upon completion of the experiment, we realized that our planned simple effects follow-up analyses for all measures would be difficult to interpret as sleep was confounded with the passage of time (length of delay). We had planned to analyze the difference between the 12-hour and 24-hour delay conditions (and the untrained condition for the semantic relatedness task) within each of the two groups. However, instead we decided that it was more informative to report the simple effect of group within each of the training conditions, which is more comparable to the analysis of Experiment 1. This is a deviation from the analysis plan outlined in the preregistration of this experiment; all of the other analyses were carried out in accordance with the preregistration. (We have included the simple effects analyses conducted in accordance with our preregistered analysis plan in the supplementary results and analysis scripts that are available via the OSF: https://osf.io/m3pj6.)

The data were analyzed in the same way as for Experiment 1. Contrasts for the two logistic LME models of the accuracy data for the cued recall and multiple-choice measures were defined using deviation coding for the fixed effects of group (AM-test group: –0.5, PM-test group: 0.5), and training condition (12-hour delay: –0.5, 24-hour delay: 0.5), with the interaction coded by multiplying the contrasts for these factors. These two models included by-participant and by-items random intercepts, a by-participants random slope for training condition, and by-items random slopes for group, training condition, and the interaction. This maximal random effects structure was simplified for the cued recall measure by removing the correlations between the by-participant and by-item random slopes and random intercepts to allow for model convergence (Barr et al., 2013).

For the models used to analyze the RT and accuracy data for the semantic relatedness judgement task, the contrast for the fixed effect of group was again defined using deviation coding (AM-test group: –0.5, PM-test group: 0.5). Contrasts for the fixed effect of training condition were defined using Helmert coding, with one contrast comparing the untrained condition with the two trained conditions combined (untrained: 0.67, 12-hour delay: –0.33, 24-hour delay: –0.33), and a second comparing the two training sessions to each other (untrained: 0, 12-hour delay: –0.5, 24-hour delay: 0.5). The interaction was coded by multiplying the contrasts for group and training condition. Both models used the maximal random effects structure that was simplified by removing the correlations between the by-participant and by-item random slopes and random intercepts (Barr et al., 2013). Inverse-transformed RTs were used for the analysis of the RT data as they met the assumptions of homoscedasticity and normality most closely.

Following on from the main analysis for the cued recall and multiple-choice measures, simple effects analyses (Bonferroni-corrected α = .025) were carried out to determine whether there was a significant effect of group within each of the training conditions. This was done by taking separate subsets of the data for the 12-hour delay and 24-hour delay conditions and creating a model for each containing only a fixed effect for group (and random effects, with a slope for group by items).

Following on from the main analyses of the RT and accuracy data for the semantic relatedness judgement task, two sets of follow-up analyses were carried out. The first set were carried out to determine whether there was a significant interaction between group and any of the three pairs of levels of training condition (Bonferroni-corrected α = .017). Secondly, three simple effects analyses (Bonferroni-corrected α = .017) were carried out to determine whether there was a significant effect of group within each of the training conditions. All data and analysis scripts for this experiment are available via the OSF (https://osf.io/m3pj6).

#### Cued recall of meanings

**Figure 7:**
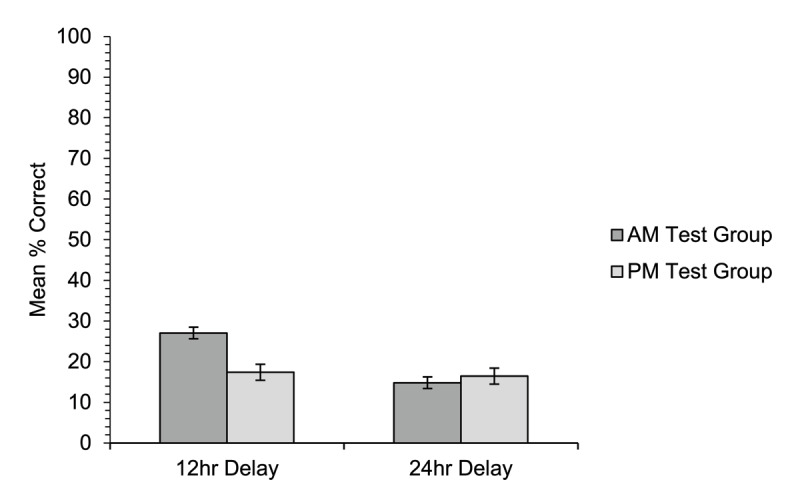
Experiment 2. Mean percentage of correct responses on the cued recall test (meanings correctly recalled for the appropriate word) by participants in the AM-test group and PM-test group for new word meanings trained either 12 hours or 24 hours prior to test. Error bars show standard errors for the means, adjusted for the within-participants factor of training condition (Cousineau, 2005).

Cued recall accuracy (Figure 7) was low overall, and did not differ significantly overall between the two groups [χ^2^(1) = 1.47, *p* = .226]. However, there was a significant main effect of training condition [χ^2^(1) = 5.10, *p* = .024], and a significant interaction between group and training condition [χ^2^(1) = 4.74, *p* = .029]. Simple effects follow-up analyses showed that, as in Experiment 1, in the 12-hour delay condition the AM-test group correctly recalled significantly more of the new word meanings (27.0%) than the PM-test group (17.4%), [χ^2^(1) = 5.12, *p* = .024]. However, in the 24-hour delay condition there was no significant difference in performance between the AM-test group (14.8%) and the PM-test group (16.5%) [χ^2^(1) = 0.01, *p* = .909; α = .025].

#### Multiple-choice meaning-to-word matching

**Figure 8:**
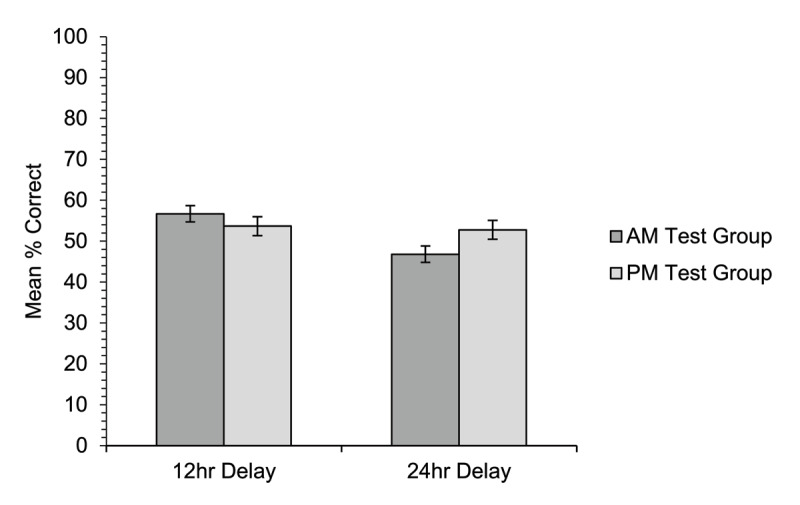
Experiment 2. Mean percentage of correct responses on the multiple-choice meaning-to-word matching test (words correctly paired with the appropriate definition) by participants in the AM-test group and PM-test group for new word meanings trained either 12 hours or 24 hours prior to test. Error bars show standard errors for the subject means, adjusted for the within-participants factor of training condition (Cousineau, 2005).

Accuracy on the multiple-choice test (Figure 8) was much higher than for cued recall, with little difference between the different conditions. There was no significant difference in accuracy overall between the two groups [χ^2^(1) = 0.07, *p* = .792]. There was no significant main effect of training condition [χ^2^(1) = 2.63, *p* = .105], nor interaction between group and training condition [χ^2^(1) = 2.46, *p* = .117]. Simple effects follow-up analyses showed that for the 12-hour delay condition there was no difference in performance between the AM-test group (56.7%) and the PM-test group (53.7%), [χ^2^(1) = 0.34, *p* = .561]. In the 24-hour delay condition there was also no difference in performance between the AM-test group (46.8%) and the PM-test group (52.7%), [χ^2^(1) = 1.24, *p* = .265; α = .025].

#### Semantic relatedness judgement

**Figure 9:**
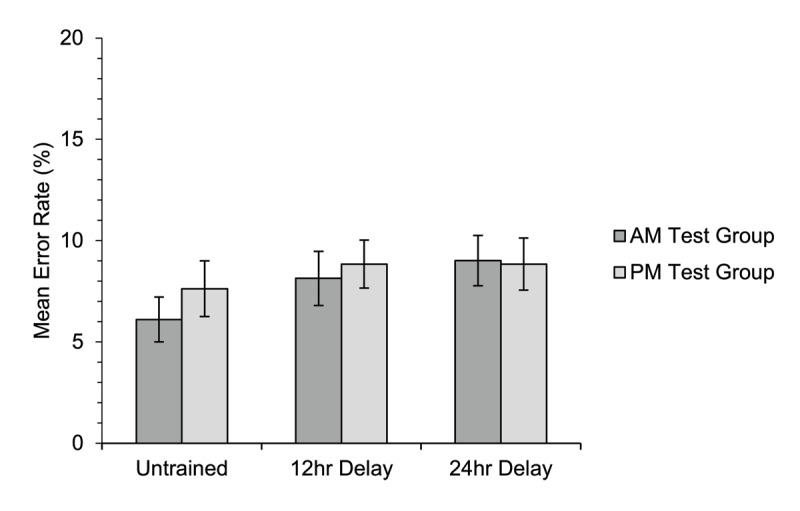
Experiment 2. Mean percentage error rate on the semantic relatedness judgement task for participants in the AM-test group and PM-test group for items that were either untrained, trained 12 hours prior to test, or trained 24 hours prior to test. The data shown are related trials only (trials in which the target and probe were semantically related). Error bars show standard errors for the means, corrected for the within-participants factor of training condition (Cousineau, 2005).

Mean percentage error rates for the semantic relatedness judgement task are shown in Figure 9. There was no significant overall difference in accuracy between the two groups [*χ*^2^(1) = 0.18, *p* = .670], and no significant overall difference between the 12-hour and 24-hour delay conditions [*χ*^2^(1) = 1.44, *p* = .486]. The interaction between group and training condition was also non-significant [*χ*^2^(1) = 0.41, *p* = .816].

Planned follow-up analyses of the three two-way interactions between group and pairs of levels of training condition showed no significant interaction between group and training for the untrained and 12-hour delay conditions [*χ*^2^(1) = 0.10, *p* = .755], the untrained and 24-hour delay conditions [*χ*^2^(1) = 0.39, *p* = .530], nor the 12-hour and 24-hour delay conditions [*χ*^2^(1) = 0.11, *p* = .746; α = .017]. The simple effects analyses showed no effect of group for the untrained condition [*χ*^2^(1) = 0.63, *p* = .426], 12-hour delay condition [*χ*^2^(1) = 0.13, *p* = .715], nor the 24-hour delay condition [*χ*^2^(1) = 0.02, *p* = .883; α = .017].

**Figure 10:**
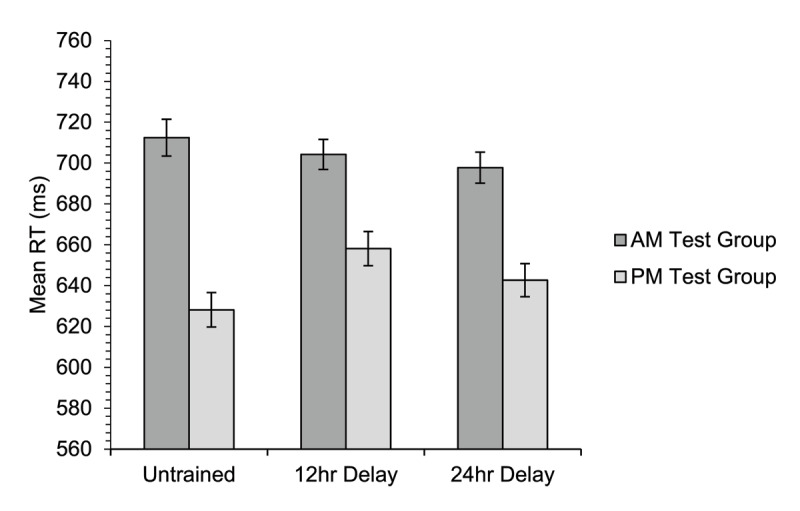
Experiment 2. Mean reaction time on the semantic relatedness judgement task for participants in the AM-test group and PM-test group for items that were either untrained, trained 12 hours prior to test, or trained 24 hours prior to test. The data shown are for correct related trials only (trials to which the participants correctly responded ‘yes’ that the target and probe were semantically related). Error bars show standard errors for the means, corrected for the within-participants factor of training condition (Cousineau, 2005).

The mean RT data for the semantic relatedness judgement task are shown in Figure 10. There was a significant main effect of group [χ^2^(1) = 5.08, *p* = .024], as the PM-test group were faster overall than the AM-test group. There was no significant main effect of training condition [χ^2^(2) = 1.88, *p* = .391], nor interaction [χ^2^(2) = 4.62, *p* = .099].

Planned follow-up analyses of the three two-way interactions between group and pairs of levels of training condition showed that the interaction between group and training for the untrained and 12-hour delay conditions was non-significant at the Bonferroni-corrected level [χ^2^(1) = 3.90, *p* = .048]. There was also no significant interaction between group and training for the untrained and 24-hour delay conditions [χ^2^(1) = 2.27, *p* = .132], nor the 12-hour and 24-hour delay conditions [χ^2^(1) = 0.19, *p* = .665; α = .017]. The simple effects analyses showed that the PM-test group were faster than the AM-test group in the untrained condition [*χ*^2^(1) = 7.14, *p* = .008]. There was no difference in RT between the two groups for the 12-hour delay condition [*χ*^2^(1) = 2.18, *p* = .140], nor the 24-hour delay condition at the Bonferroni-corrected level [*χ*^2^(1) = 3.84, *p* = .050; α = .017].

As for Experiment 1, we carried out additional exploratory analyses of the semantic relatedness judgement data, with a subset of only trained items for which participants had correctly recalled the item in the cued recall test. Results were largely similar to the main analyses (see Supplementary Results: https://osf.io/p3t49).

### Discussion

The aim of Experiment 2 was to further examine the benefit of sleep for learning new word meanings that was seen in Experiment 1 by testing participants both 12 and 24 hours after learning. The cued recall measure showed a significant interaction between training condition and group. Importantly, the simple effects revealed that when tested 12 hours after training, performance was better for the AM-test group who had learned the new word meanings the night before (immediately preceding overnight sleep) than the PM-test group (for whom this training session preceded a period of wake). This directly replicates the finding from Experiment 1. In contrast, when tested after 24 hours we found no effect of the relative timing of participants’ sleep during this 24-hour period: contrary to the prediction of the active consolidation account, the PM-test group did not show better recall accuracy than the AM-test group, despite this training session being immediately followed by sleep for the PM-test group. In contrast to the clear pattern of results seen on the cued recall task in Experiment 2, neither the multiple-choice recognition task or the speeded semantic relatedness task revealed significant effects of either training or group. It is unlikely that that the significant effects on the cued recall task are simply due to circadian effects due to the different times of test for the two groups. This explanation would predict a straightforward pattern whereby improved memory of the new word meanings would depend on the time of day at which participants were trained or tested. The results of the cued recall measure are not consistent with this explanation, as the AM- test group did not perform significantly better overall than the PM- test group. The results therefore cannot be explained in terms of a time of day effect or general cognitive enhancement improving recall function (Schmidt et al., 2007). There was also no significant difference in SSS rating between the two groups at either of the training sessions or the test session. Furthermore, attrition from the two groups was more balanced in Experiment 2, which replicated the finding from Experiment 1 in the cued recall task; the results of Experiment 1 are therefore unlikely to be explained by selective attrition.

## General discussion

The present experiments explored whether sleep is important for learning new meanings for familiar words. Experiment 1 found that participants had better recall and recognition of new meanings for familiar words when training was followed by a 12-hour period that included sleep compared to a 12-hour period of wake. This observed sleep benefit after 12 hours was replicated in Experiment 2 on the cued recall measure, but was not significant for the multiple-choice recognition task.

Given the clear effect of sleep on this task in Experiment 1, it is unclear why this recognition task was less sensitive to training effects in Experiment 2. This pattern is however consistent with previous work that has suggested that sleep benefits are more consistent for recall than recognition measures (Berres & Erdfelder, 2021; Diekelmann et al., 2009; Mak et al., 2023; although see Schimke et al., 2021). This is may be because sleep-associated benefits are stronger for more weakly encoded memories (Denis et al., 2021; Schoch et al., 2017); cf. Walker et al., 2019), and cued recall is a more sensitive measure to capture quality of vocabulary knowledge than recognition-based measures like multiple-choice. In addition, the repeated testing of the same items at test may have attenuated effects on the recognition test, which participants performed last and that, perhaps in combination with the more extended experimental design used in Experiment 2, reduced the sensitivity of this measure. Despite the absence of a significant effect on this measure in Experiment 2, taken together the results of the two experiments provide clear evidence that word-meaning learning is enhanced after a 12-hour period that includes sleep compared with 12 hours of wake.

However, this 12-hour sleep benefit may not reflect active overnight consolidation processes. The sleep benefit may instead reflect more passive protection from interference that arises from the linguistic input that participants likely encounter during a typical 12 hours of wake (Ellenbogen, Payne, et al., 2006; Rasch & Born, 2013). Experiment 2 therefore extended this 12-hour paradigm to test participants’ knowledge of learned word meanings after 24 hours. Not only did this design examine the persistence of the sleep benefit, but it tested the prediction of the active consolidation account (Davis & Gaskell, 2009) that 24 hours after learning performance should be better in the PM-test group compared with the AM-test group. This difference was predicted to arise because the PM-test group had a period of sleep immediately after learning that could support active sleep-related consolidation processes that would protect from forgetting during their subsequent day awake. In contrast, the AM-test group learned the meanings immediately before a period of wake and so would likely have (partially) forgotten much of what they learned before they had the opportunity for overnight consolidation.

Surprisingly, the results from the cued recall test did *not* support the active consolidation account. Although we observed the predicted interaction between group and delay, the simple effects indicated that this interaction was driven by the significant group difference after a 12-hour delay. The predicted group difference after a 24-hour delay was *not* observed (see Figure 7): there was no benefit for participants who were trained immediately prior to sleep and tested 24 hours later. This pattern of results is fully compatible with the view that the 12-hour sleep benefit reflects a transient form of protection from interfering linguistic information (Ellenbogen, Hulbert, et al., 2006). Gaskell et al. (2019) suggest that if the benefit of sleep is due to passive protection from linguistic interference, then 12 hours of interference before sleep would have the same detrimental effect as 12 hours of interference after sleep. If this passive account of the 12-hour sleep benefit is correct, then protection against interference from new linguistic input would likely provide only a short-lived boost that will disappear as soon as participants spend a day awake. This account predicts limited real-world/long-term advantages for words learned immediately before sleep, as these would be unlikely to endure beyond 12 hours. This is in contrast to research that has found a vocabulary learning advantage for stories read around bedtime (Henderson et al., 2015, 2021), albeit with different training and testing schedules than in the present study.

Importantly, although the results from the current paradigm seem more compatible with the ‘passive protection’ account, we caution against interpreting them as *incompatible* with active consolidation (Ellenbogen, Payne, et al., 2006). Specifically, the absence of a group difference in the 24-hour conditions of Experiment 2 does not provide strong evidence against this theoretical account. There are several reasons that this null finding might have emerged even if the 12-hour sleep benefit was due to active consolidation. Firstly, the items trained 24 hours prior to test could have suffered from specific interference from items trained later in the 12-hour delay condition, reducing the opportunity for subsequent consolidation. Secondly, the sleep benefits of passive protection and active consolidation are not mutually exclusive. We therefore must consider the possibility that the 12-hour sleep benefit is a result of additive effects.

The passive account of the benefit of sleep remains somewhat underspecified with regards to the factors that might modulate interference from linguistic information during periods of wake, and the timing of learning relative to subsequent periods of sleep. For example, spaced learning throughout the day (Lindsay & Gaskell, 2009, 2013) and repeated testing (Antony et al., 2017) have both been suggested to counteract interference and may provide a fast track to memory consolidation. There are also some mixed findings regarding the timing of learning before sleep: some research has found a benefit of post-learning wake prior to sleep for longer-term retention (Alger et al., 2010; Walker et al., 2019).

Finally, although the results from the explicit memory tests discussed above provide clear evidence of sleep effects, no effects of either training or group were observed on the speeded semantic relatedness task in either experiment. In contrast to the explicit memory tests, this task, which was first used by Rodd et al. (2012), was designed to reveal any effects of learning new word meanings on participants’ existing lexical knowledge. For example, we tested whether learning a new meaning for a familiar word form like “foam” would impair their ability access this word’s familiar meaning and judge whether it was related to the probe word (e.g., *foam-soap*/*foam-belt*) (Fang et al., 2017; Fang & Perfetti, 2017; Maciejewski et al., 2020; Rodd et al., 2012). This task was designed to be analogous to studies of word-form learning which measure the impact of learning a new word form (e.g., “cathedruke”) on a familiar word form (“cathedral”) (Gaskell & Dumay, 2003; Lindsay & Gaskell, 2013). As discussed in detail by Leach and Samuel (2007), improvements in explicit memory performance alone should not be taken as evidence that newly learned information about words has been consolidated into long-term lexical knowledge without corroborating evidence from implicit memory measures that assess the impact of lexical learning on pre-existing lexical knowledge (Fang et al., 2017; Fang & Perfetti, 2017; Maciejewski et al., 2020; Rodd et al., 2012). There are two, related explanations for the consistent null findings on this task. First, we may have lacked sensitivity to detect competition effects due to relatively weak learning of the new meanings, as evidenced by the low performance overall on the explicit measures of word meaning knowledge, perhaps due to the incidental learning procedure (see Hulme & Rodd, 2021). Previous studies that have shown robust effects of new meaning learning on similar, speeded tasks have typically used more intensive multi-day intentional training paradigms (Maciejewski et al., 2020; Rodd et al., 2012). One way to potentially boost learning whilst retaining the naturalistic training paradigm would be to increase the perceived relevance of the information for participants by creating the expectation that they would require this knowledge to support comprehension of follow-up chapters the following day (Gong & Rodd, 2020). Second, the null findings may reflect low statistical power. Measures of lexical integration typically show smaller effect sizes for the benefit of sleep than explicit measures of recall and recognition (Schimke et al., 2021), and participants only learned eight meanings per session. Taken together these factors mean we should therefore be highly cautious when drawing inferences from these null findings.

In summary, the current experiments demonstrate that explicit memory for newly learned word meanings is enhanced when tested after a 12-hour period that includes overnight sleep compared with wake. However, this group difference was absent when participants were tested 24 hours after learning. These results are fully consistent with the view that the initial sleep benefit arises due to passive protection from the linguistic interference that usually occurs during wake. In contrast, the observed pattern is less clearly consistent with the active consolidation account (Davis & Gaskell, 2009). However, in the context of the growing number of studies linking sleep directly to the consolidation of new word forms (e.g., Dumay et al., 2005; Tamminen et al., 2010), and new words and their meanings (e.g., Clay et al., 2007; van der Ven et al., 2015), we urge caution in over interpreting the current results as strong evidence against the active consolidation account. Future studies should include polysomnographic recordings in combination with behavioral measures to more directly probe the association between integration of new word meanings into semantic memory and specific components of sleep that have been linked to memory consolidation (Tamminen et al., 2010).

## Data accessibility statement

Our materials, data, and analysis scripts are available on the Open Science Framework: https://osf.io/m3pj6. Experiment 2 was preregistered: https://osf.io/uvgp4.

## Additional Files

The additional files for this article can be found as follows:

10.5334/joc.282.s1 Supplementary Materials. Stories.

10.5334/joc.282.s2 Table S1. List of stimulus words and definitions of their meanings.

10.5334/joc.282.s3 Table S2. Stimulus words and paraphrased versions of their definitions.

10.5334/joc.282.s4 Table S3. Target-probe word pairs used in the semantic relatedness judgement task.

10.5334/joc.282.s5 Table S4. Properties of the probe words in the semantic relatedness task in Experiment 1.

10.5334/joc.282.s6 Table S5. Properties of the probe words in the semantic relatedness task in Experiment 2.

10.5334/joc.282.s7 Supplementary Results. Alternative Simple Effects Analyses.

10.5334/joc.282.s8 Supplementary Results. Exploratory Analyses.

## Footnotes

Analyses of pilot data indicated that it was not possible to use a more graded measure of the number of semantic features correctly recalled (e.g., Hulme et al., 2023) due to the naturalistic nature of the training materials. The number of semantic features was not explicitly held constant across items in the present experiments, in favour of embedding the novel word meanings in naturalistic story narratives.

For one item (“foam”) accuracy was slightly lower than three *SD* below the grand mean for all items (89.88%), however as it was only 0.29% lower the item was kept in.

We included a factor for block for the semantic relatedness task due to a potential order effect whereby participants’ responses might have differed depending on whether they had already seen a given target word with its unrelated probe word (‘no’ trial) in the previous block.
