# Supplementary material for: The Role of Sleep in Learning New Meanings for Familiar Words through Stories
Source: J Cogn. 2023 Jun 15;6(1):27. doi: 10.5334/joc.282 (PMC10275344; doi:10.5334/joc.282)
Supplement: Supplementary Materials. — Stories. [file joc-6-1-282-s1.pdf]

 Copyright: CC-By Attribution 4.0 International

Supplementary Materials

The four stories that were used as the stimulus materials. The stimulus words have been underlined for transparency, but were not highlighted in any way when presented to participants in the experiments.

Story 1: Pink Candy Dream

The minute I pulled open the door and squeezed myself in behind the wheel I knew that Marla had borrowed the hive to take Lilly to playgroup again; the side pockets were bulging with plastic animals, baby wipes and half-eaten breadsticks. I extracted a neon pink sippy cup from the drinks holder and replaced it with my double shot Americano and wedged my kit bag into the hive's tiny passenger seat on top of a fleecy jacket and a sparkly pink slipper. Not for the first time, I was struck by a pang of longing for the old days - when cars came with luxuries like a boot, a back seat and more than three millimetres of leg room.

'Downtown! Quadrant Three!' I instructed the dashboard.

The electrics flickered on and the hive eased out into the flow of traffic. We climbed quickly to the top of the six road levels, squeezed between a pair of commuter buses and darted into a side street. As we zipped past nightclubs, betting palaces, sugar dens and bingo halls, I wondered how many of them had been taken over as vases; establishments used by the big criminal gangs for their clandestine deals and deliveries.

'Dammit!' I groaned, wiping a smear of mashed banana from my sleeve.

'Hey, Mike, you sound a little tense. How about we swing by the mall for a bubble tea? Wouldn't that be awesome?' The hive's digital voice was that of a relentlessly chirpy Californian teenager with a brand of American slang, which the car's Chinese designers must have picked up from old episodes of *The Simpsons*. 'Or what say a relaxing head massage?' the hive persisted.

'Just shut up and drive!' I snapped. When I was a kid, equipment was seen and not heard; it didn't pipe up with 'useful' suggestions every five minutes.

Don't get me wrong. I'm not usually one of those *everything-was-better-in-the-old-days* kind of a guy. Who'd want to go back to a time days before we had decent hair implants and a cure for dementia? And the hive could scurry through midtown traffic like nothing else. My bad temper was a stress thing, that's all. I'd been summoned for a "breakfast meeting" with Control. And Zinnia Mendez wasn't the kind of boss who called her operatives in for chitchat and croissants.

***

 Copyright: CC-By Attribution 4.0 International

[SCREEN PAGE BREAK]

'How has this happened?' Controller Mendez demanded as I entered her corner office. She was staring out of the floor-to-ceiling window and didn't turn round. Instead she spoke her words to the pane of tinted smart glass and the muddy sky beyond. 'I *thought* we were keeping the Olafson gang under 24/7 watch?'

'We are,' I told the back of her jacket. The golden sheen suggested it was the latest spider silk microfibre. 'We've had nests of paths monitoring all their active vases for weeks,' I said, referring to the minute bugging devices that used the latest in nano-communications technology. Invisible to the naked eye, they were suspended in an adhesive solution and sprayed directly onto walls and ceilings.

Mendez didn't speak. A Control helicopter buzzed past the window.

Still standing, I gulped my cold coffee. The paths were my baby. It was like having thousands of tiny eyes and ears relaying messages back to Control. *Had something gone wrong?*

A very large number flashed before my eyes; the annual cost for the exclusive school we'd signed Lily up to. I couldn't afford to lose my job over this . . .

Mendez whipped round and leaned over her granite desk until her face was so close I could smell her lipgloss. *'They are one hundred percent undetectable!*' she mocked in a whiny voice, which seemed intended as an imitation of my own. *'Those Olafson scumballs won't suspect a thing . . .'*

'They are,' I spluttered. 'They won't.'

'So how do you explain *this?'* Mendez snarled. She pushed back from the desk and clicked her fingers. A virtual screen appeared between us and began to play surveillance footage.

The room was familiar. I'd installed the paths there myself. The walls were panelled in genuine antique pine and the floor carpeted in synthetic tiger skin. It was a lot swankier than the usual pool halls and strip clubs that the Olafson gang used as their 'business vases' for dropping off consignments of drugs and other illegal goods and picking up money. This was Olafson's personal penthouse apartment on the lagoon. It had been a nightmare to get in and out past security.

On screen a tall, thickly-bearded man entered the room. I recognized Olafson from a previous vase bust. He glanced around before flopping onto a yellow velvet sofa. He held up a small device and rotated it above his head.

'Is *that* what this is about?' I almost laughed with relief. It was common knowledge that all the gangs used portable scanners – nicknamed Rentokills - to make sure their vases were free of bugs and cameras. Obviously, I'd made sure that paths couldn't be picked up by any of the Rentokill models on the market.

 Copyright: CC-By Attribution 4.0 International

Controller Mendez skewered me with a look. 'Just keep watching.'

[SCREEN PAGE BREAK]

Olafson was now peering at the armrest at the end of the sofa. It was one of those old Victorian types - with an arm at just one end – that are all the rage these days. *Chaises-longues,* I think they're called. Beneath the velvet cover, the armrest was polished wood, fashioned into an ornate scroll shape. As the camera feed zoomed in closer I couldn't help a moment of pride in my design – the paths use artificial intelligence to cluster into the best position and they work together to fine-tune the signal. Now they were magnifying the hairs on the back of Olafson's freckled hand to a forest. His fingers became those of a giant. A glint of silver revealed that they were curled around a delicate key. I held my breath as he slid the key into the centre of the scroll. A section sprang open and he pulled out a long, engraved tube.

'Yes,' I murmured, breathing again. 'I *knew* it! It's a foam**.**' The ornate *chaise longue* was no ordinary piece of furniture, but concealed a built-in safe with an intricate key-operated locking system.

I looked up at Zinnia Mendez. Her mouth was still knotted into a scowl. I couldn't figure out why she was so ticked off. 'Isn't this exactly what we've been looking for?' I asked. 'If we can find their foams we can find their paperwork . . . and bust them!'

'There's the small matter of *unlocking* the foams first, Michael!'

The voice came from near the door. I span round, my heart thumping in my chest. I hadn't heard anyone come in. An angular man in plain black uniform moved noiselessly towards the desk, his small feet seeming to slide rather than walk across the floor. Mendez registered his presence with a twitch of her eyebrow but didn't introduce us. I assumed he was from Internal Investigations. He narrowed his pale eyes in my direction. 'The foam's useless without knowing where Olafson keeps his keys.'

Now tell me something I don't know! I stopped myself saying the words out loud. You didn't want to get on the wrong side of Internal. I closed my eyes. Maybe I *am* one of those guys who thinks everything was better in the old days; it was so much easier when we just had to break computer passwords and decrypt files. But – like most of the serious crime rings - Olafon's gang had given up doing business on the internet. Hackers – on both sides of the law – had got so good at breaking down digital security that it was too dangerous. There was nowhere to hide. The gangs were all moving out of cyberspace, leaving it to the pornographers and petty scammers. The big money – drugs, weapons, tax fraud – had gone off-line.

Nowadays they kept their records on *paper.*

[SCREEN PAGE BREAK]

 Copyright: CC-By Attribution 4.0 International

At first they'd stored their client lists and dodgy contracts in regular safes, but when those had become too easy a target, they'd moved on to less obvious hiding places. Foams were the latest trend – small cavities concealed in the frames of seemingly ordinary pieces of furniture. It had become something of a status symbol to commission bespoke cabinets, bureaus, love seats and *chaises longues* with the most cunningly hidden and exquisitely complex locking mechanisms. Skilled locksmiths could name their price. Hiding the tiny keys had become an art in itself; dentists had been known to hollow out teeth.

I turned my attention back to the screen. Olafson had unfurled a scroll of paper from the tube. He placed a little wooden clipboard on his lap, took a fat glossy fountain pen from his shirt pocket and began to write, his tongue sticking out from the corner of his mouth as he formed the letters. He blotted the ink with a cloth – he was clearly one of those gang bosses who'd taken retro writing to the extreme.

I was still trying to figure out why I was being hauled over the coals when Mendez clicked her fingers and told the film to move forward one hour.

Olafson had left the room. Workmen had arrived and were starting to move the furniture out and take up the carpet. 'What's going on?' I breathed, as they lugged the *chaise longue* towards the door. Surely Olafson wasn't offloading his foams already? It was one of our biggest problems. Criminals never kept the same pieces of furniture for long. They were always lusting after the latest high-status foam designs. Which meant *we* were always playing catch-up.

On the film, two young men in white disposable overalls chatted about the latest 5D computer game while they attached pipes to a pair of large metal canisters. They fiddled with the nozzles for a moment and then - pretending they were gunning down an enemy attack - aimed them at the walls. There was a pink blur. A series of different camera shots kaleidoscoped round the screen as the paths in other parts of the room tried to fill in the lost signal. The sound broke up and was replaced by a static hiss. Within minutes we were looking at a blank screen.

Something *had* gone wrong. Very wrong.

'That's the whole operation gone to waste,' 'Controller Mendez spat. 'Olafson's twigged that we're watching him.'

'Maybe there's another explanation . . . ' But I was just playing for time.

The Controller slapped her hands down on the desk. 'He has all the furniture removed. Then he sprays some kind of *paint* over the walls to block out the paths**.** What other *explanation* is there?' She was shouting now. Specks of spittle peppered the lapels of her spider silk jacket. 'He knows we're on to him. All the foams will have been emptied. He's probably cleaned out his entire network of vases already.'

 Copyright: CC-By Attribution 4.0 International

[SCREEN PAGE BREAK]

As Mendez ranted my brain was working overtime. Something on that film just didn't add up. Those workmen didn't look like gang members; the sort that Olafson would entrust with a big security lockdown. They'd looked – and acted – like regular labourers on an everyday decorating job. And there was something else tugging at my memory. Something had made me think of Lily; her things all bundled into the side pockets in the hive, her pink slipper on the seat, her pink sippy cup.

And suddenly I had the answer. *Pink!*

'Olafson's married, right?' I asked.

Controller Mendez glared at me but the man in black nodded. 'Yeah. Lucetta Stone. She was a lap dancer in a club the gang took over as one of their first vases when they were just starting out.'

'Kids?' I asked.

The man shook his head. 'We think she may be sick. She's been visiting a medical clinic on Riverside . . .'

'It could be a new vase.' Mendez interrupted.

The man from Internal Investigations shook his head. 'There was no sign she was meeting a contact there. No drop-offs. No deliveries.'

'So she's having a boob job or a laser tuck?' Mendez snapped. 'What's the point of this?'

'Go back to the last frame,' I said, talking to the computer screen. It didn't even flicker. Its voice recognition couldn't deal with my British accent. For once I missed the hive's digital teenager. At least she listened to me.

Mendez sighed, but then turned to the screen. 'Back!' she said, flicking her hand to show how far.

'Stop! That's it!' I cried. 'Look!' The frame froze on a view of the paint canisters. It was blurry – one of the last shots before the paths were blotted out.

Mendez told the computer to zoom in, and there it was. The name of the brand of paint was printed on each canister; *Lullaby Magic*. The colour was labelled too; *Pink Candy Dream.*

I was right!

Lullaby Magic was the best money could buy. The paint had a luminescence that supposedly sparkled in synchrony with the human heartbeat. It was also suffused with soothing tones of vanilla and chamomile for 'unbroken nights of blissful slumber.'

 Copyright: CC-By Attribution 4.0 International

We'd only been able to afford one tiny wall of *Pink Candy Dream* when we decorated Lilly's room.

'I think that Olafson and his wife *are* expecting a delivery at the medical centre.' I said. 'A very special kind.'

Zinnia Mendez and the man in black wore matching blank expressions. I'd have bet my savings that neither had children. 'They haven't rumbled our surveillance,' I explained. 'They're turning that room into a nursery.'

I just hope the Lullaby Magic is more effective on their baby, I thought. 'Blissful slumber' had become something of a family joke between Marla and me. Although at four in the morning neither of us ever found it very funny.

 Copyright: CC-By Attribution 4.0 International

Story 2: Prisons

When they put this pacemaker inside my chest I thought that – well I thought, I thought at least I'd be exempt now; the radiation of the fuel they load into their ships, the magnetic flux of Interspace travel, the half-dead waves still emitted by the hard, petrified star itself. Each of these things would normally shut down, short-circuit and destroy a pacemaker. No, they told me, no, that won't be a problem. And so they opened me up again and the biomedics worked their obscure science and they fixed me up with a protective dawn to act as a guard for my heart, for my pacemaker. This will protect you, they said. This will guard you: the electromagnetic signals will not be able to permeate the dawn; your pacemaker will be safe. They explained it all with words and phrases I could not understand. Well I could not argue with that, and so they shot me off into space along with all the other criminals too low-down to live on Earth any longer.

As a resident of a maximum security prison I was treated as falling *below* the net of society. My crime was so horrific, they tell me, that even I cannot now remember it. They ensured that. Every record of my crime has been wiped, and the military-trained biomedics that served as our Guards have eradicated the event from my mind. Society has licence to ignore me completely; everyone knows that a maximum security inmate belongs to a different order of humanity, that they can experience a sanctioned revulsion at the very thought of us. All of this we know. I know that during my time in maximum security I could not be considered a human, not in the true sense. I accepted this, I still accept this, just as we all accept it.

And this is how they sold us the idea of the mining expedition. The Guards gathered the worst of us in a large well-lit hall, which burned our eyes after countless weeks in the darkness of our cells. The room was filled with charts and maps and projections and a hologram model of a crusty white orb, floating and rotating freely in the centre of the room, about the size of a small transport vehicle. One of the lads pointed to the orb and shouted: 'now that's one pill you biomeds ain't gettin me to swallow.' We all laughed at that, but the Guards did as they always did – they ignored the joke completely, ignored our laughter, until the vacuum of attention became so oppressive we all slowly stopped and looked at each other with something approaching shame.

[SCREEN PAGE BREAK]

'Take a seat' said a woman in a uniform none of us could quite place. And so we all sat around the table that this orb was hovering above. The Guards let us sit in an uncomfortable silence for a long moment while they looked over the files contained on their tablets. One senior biomed emitted a series of short tut-tutting sounds and shook his old head. He was the first to look at us. 'You're all making us look rather bad' he began. 'Do you know where we are?' He looked around at our faces. 'Do you?'

'In a maximum security prison, sir' said one of the younger men.

 Copyright: CC-By Attribution 4.0 International

‘That’s right, that’s absolutely right’ the biomed said over the brim of his glasses. ‘And the point of a maximum security prison?’

‘To keep us all away from – from the outside, sir, from society, sir.’

‘Exactly right; to keep all of you *outside* of society. But you lot are *notorious* aren’t you?’ We did not know, could not remember anything, and yet we accepted this piece of information as we did all pieces of information in those days. ‘Yes, you are all *well-known*. And this poses a problem for us. People cannot forget you as they ought, even with you in here there are those out there who still talk about you, about all of you, in one form or another, and this will not do. We need you *removed* from society… *entirely*.’ He closed his eyes for a moment and sighed. ‘But never mind that. We have a solution. Yes, do not worry about that. We have found a way to make you all – become forgotten, soon enough. Don’t you worry about that.’ He smiled and met our gaze. ‘We have an exciting proposition for you all.’

A Guard officer pointed to the large spinning white orb. He explained that what we were looking at can only be seen from the most advanced telescopes in society. The kind owned only by the most elite institutions. In fact, he explained, until a very short while ago we had no real idea of what it was at all. Well, now all of that had changed. What we were looking at was death. It was known as a <u>spy</u>, and, it was, as he read from a tablet he held in front of his chest, the unique, hardened remains of a supermassive star – a star which had not become a proud black hole, but which had simply become too large too quickly, had expanded into nothing, and had left behind only a dry core to wither and cool. We were looking at a <u>spy</u>, that’s what they called it, and we were going to mine it.

[SCREEN PAGE BREAK]

They showed us all pictures of men and women in their full-body <u>feasts</u> – necessary wear to protect oneself from the radioactive dust thrown up by the mining process. From the singular eye-slit in one man’s <u>feast</u> it was just possible to make out the edges of a smile. The miners are all treated very well, we were told. Each of them is free to do as they please on the satellite station orbiting the <u>spy</u>, during their time off. Each and every worker up there, in deep space, mining, is making a singular and worthwhile contribution to society, and are therefore integrated deeply *into* society. This we were told. For many of us this seemed like an impossible dream. We knew we were not accepted as part of the world here in our prison. But out there, in the frozen pitch of space, we could finally contribute to society and to become a part of it again. Well. I was sceptical.

It was around this time in my life that the biomeds diagnosed my heart condition. They regulated my failing heart with a pacemaker. They cut me open and put this thing inside of me. They told me I wouldn’t even notice it was there. That was largely true until they cut me open again and put a <u>dawn</u> over it. The reason for the <u>dawn</u> was to ensure I could make the Interspace expedition to the mining

 Copyright: CC-By Attribution 4.0 International

colony, to ensure that my pacemaker did not fail at the first whiff of the spy radiation. Yes, the device was my liberator from the maximum security prison, even if it liberated me for an expedition I did not want to take.

Many of the lads were pretty excited about the whole thing. The day before we left, they gathered us all in to this little white room with medical equipment hanging from the walls. Everyone was whispering their nervous anticipation at the idea of mattering again. And what they told us all then really cemented that sentiment, and everyone felt very good about the whole thing. They told us we would each have to wear a pearl - they wrapped this device around our left biceps, and informed us that they would remain there, under our feasts, for the duration of our expedition to the mining colony. They explained that it would innocuously, without piercing through our skin, unobtrusively, monitor everything it could about us (which, they said, was a great deal more than only a few years ago) via our blood. The information from the pearl would then be sent back through Interspace-waves and arrive safely with someone somewhere back here. We didn't need to worry too much about that, they said, so we didn't. The pearl was quite plain and unassuming and I could barely feel it clinging to my arm, so I really didn't need to give it much attention (in fact it is only now, recounting this part of my tale, that I have given it any thought whatsoever). I could see the faces of the men in the room, their pearl worn proudly around their arms, and I could almost see their tiny, shrivelled, ignored and neglected identities building themselves up again, slowly, knowing that someone, somewhere, was taking an interest in them.

They never actually explained why we had to wear these pearls, but none of us asked so I suppose it was we who were in the wrong. We really should have sensed something when they put these things on us. We should have known, really.

[SCREEN PAGE BREAK]

We took off the next day. They performed a few last-minute checks on all of us. They took us away, one by one, for privacy's sake, into a small medical room and gave us all a once-over. When my turn came, they took me in and placed two electrical prongs over my chest, testing, they told me, that my dawn was in perfect working order. It was, and I was cleared for take-off.

The shuttle was quite sizeable, and after we had left Earth's orbit, had punctured Conventional space and had slid into Interspace, we were able to move freely around. The expedition would take about a week, they had said. During that time we would all be required to wear our feasts, without the helmet, to protect us from the worst of the high radiation of Interspace. 'This thing is intolerable!' shouted one of the men on the ship. He was complaining about the fact that our feasts were intolerably scratchy. He was complaining that our feasts were intolerably tight in all the wrong places. 'Intolerable, really uncomfortable, I can't wear this thing' he said, as he ineffectually pulled at the suit. We all looked at him then, looked around the sizeable cabin in which we were free to move around, considered the

 Copyright: CC-By Attribution 4.0 International

endless free flux of Interspace we were moving through, thought about the mining colony with its meaningfulness and free time. We all looked at him thinking about these things and he saw those thoughts in all of us. That made him quiet down, and no one complained about the really very uncomfortable (and intolerably itchy) suits again.

On the third day I started speaking to one of the other lads on the ship. I pointed to a scar on his head. 'What's that scar on your head all about?' I said.

'Oh this?' he pulled off the glove of his feast and tapped his temple with his finger. 'This is from where they had to implant my, my – my regulator, you know? For my brain. Apparently I am very susceptible to, to, to strokes. I could suffer one any minute, they say. So they had to put this thing in to stop all that.'

'Right,' I said.

'Yeah. It's lucky they diagnosed me in time.'

'Oh yeah?'

'Yeah, they only installed this thing just before we left. One week later and I'd be up here ready to blow a fuse any second.'

'That's lucky,' I said. I felt anxious.

'Well they had to put this other thing in there too.'

I unconsciously grasped my chest.

'Yeah, to stop the radiation or flux or something. They called it – something – they called it – '

'a dawn?'

'That's right. That's what they called it.'

[SCREEN PAGE BREAK]

Well it turns out that we all had one of these things inside of us, for one condition or another. Each of the men (I spoke to them all in turn, quizzed them) had a condition, a device to cure it, and another device, a dawn, with which to protect it. My heart was probably fine, is probably fine, and yet I still have this pacemaker and this other thing inside my chest. And the pearl? I don't know what that blood-monitoring pearl was all about, come to think about it.

I do know how things are now.

 Copyright: CC-By Attribution 4.0 International

When we arrived at the spy we were shown around by someone who kept glancing at his watch. After a few days we saw very few people on the spy's crust. Very few miners. Most of them, we found out, were doing nothing at all. Just wandering around. Eventually we all worked out that none of us actually had to do any mining at all. None of us had to do anything. There were some who would pull on their feast every artificial morning and go out mining, of course. But I do not talk to those people any more. I have not done so for quite some time.

Up here, with my pearl still monitoring everything I do (maybe I should take it off?) I am reminded of something we were told all those years ago in the briefing room with the floating spy. This place can only be seen, they said, from the most advanced telescopes on Earth, owned by only the most elite institutions. Well the only institution which could be considered remotely *elite* is the same one which owns the maximum security prison, which owns everything. Well. It is hard not to feel watched when you think about that.

I can't say I know why I'm here: I don't. Maybe I'm some kind of test subject, maybe I'm not. But I have a funny feeling I'm being watched. Or: I am being watched by some, while others forget. What interest anyone ever had, ever really had, in this spy, or the dawn around my pacemaker, I will never know.

I suppose I know very little, now I come to think of it.

 Copyright: CC-By Attribution 4.0 International

Story 3: Reflections upon a Tribe

The Elder, when he appears, is wearing the ring. He holds his staff aloft and all can see the intricate brass weave on his finger, constricting the small gem which fades through myriad colours. The musicians of the all-male tribal folk band, known as the bruise, stand by the elders of this small tribe, solemn and still, knuckles white wrapped around their rudimentary wind instruments. They patiently await their turn to perform the ceremonial songs of old on their soft-sounding wooden flutes and sheepskin drums.

Stepping forwards against the heavy blue sky, the Elder leads the rather intoxicated villagers (we have all partaken of a potent brew), the dancers, and the musicians of the bruise themselves up to the flatrock overlooking the plain. The sombre group winds slowly up the hillside, and just for a moment one may catch a glimpse of the Elder almost as Abraham himself, leading his only son to his sacrificial death on the mountaintop. I do not know what to expect on the peak, on the flatrock, but the faces of those I have studied now for five long months tell me enough.

The weather is coming in now, and the wind whips up the long dyed gowns of the dancers walking amongst the villagers. Looking up from the base of the winding track, in the deep gloom, the entire tribe appear to comprise a singular dancer; the fluid movements of the tribe's ascent uniformly inimitable. In the daylight, under a clear sky, in the village down here in the lowlands, the street-performers dance an exuberant dance – the fog. The dancers elongate their bodies and sway in fluid motion from side to side, but keep the head still and undisturbed. The fog is one of the many charming qualities, and there are many, of these people. Now, however, under a dark sky heavy with rain, the winding tribe are led by a man disturbed, creeping ever further up the hill in an inverted reflection of the jubilant dance.

[SCREEN PAGE BREAK]

I stay behind awhile on the pretence that I wish to photograph the ascent. This itself is taken with scepticism by the villagers, but the Elder speaks some words I cannot yet grasp and I am tolerated. I assemble and properly arrange my camera, load the film, and take cover underneath its black shroud, holding the trigger ready under my thumb. I hesitate as I watch the familiar fog dance unfold at half speed through the lens. And then for a curious moment, I feel a coldness in my heart. I feel gripped by the impression that I really ought not to take the picture of the fog dancers. I become fearful, anxious. I stare for several seconds, breathless. Somehow my thumb comes down and the loud click of the camera removes me from my reverie.

I uncover myself and turn swiftly away from the procession of villagers, bruise-men and fog dancers, being led towards the summit by the Elder. I shall have the film destroyed, I find myself thinking. The photograph is not, however, my true reason for the delay in climbing the peak.

 Copyright: CC-By Attribution 4.0 International

Sure that I cannot be seen, I run through the wet grass to my tent. Once there, I begin hastily preparing my scientific instruments for travel. I mean to leave promptly after the ritual on the flatrock; the women will immediately begin the hunting season. The men shall retire to their lodge (the location of which is still a secret to me) for the old, long initiations of the sons of bruise-men into the revered band. It is a strict tribal tradition that when a player retires his closest living male relative should take over his position, which is considered a great honour.

At this point I shall be able to slip away. I must leave. The general feeling appears to have shifted of late. Where once I was warmly welcomed into this small tribe, an aura of anxiety and suspicion has grown malevolently on the horizon. In my dreams these tribespeople, who I must remind myself in my waking hours are little more than savages, become my tormentors. The bruise, who play peaceful folk music on soft wooden flutes and sing of nature in the sun now play an eerie tune and scream pagan incantations in the darkness of my sleeping mind, while the fog becomes not a charming dance, but a tormenting and disturbing display.

My jars clink and test tubes rattle as I swiftly wrap them in napkins and handkerchiefs for the long journey I must retrace through the lowlands. Almost packed, I turn my attention to a small wooden box on the earthen floor. Yes, why not, after all? I think I may permit myself one last experiment.

I open the lid, pull out the concertina of compartments on either side and prop up each of my measuring devices. I unfold a small stretch of paper with my graphs and annotations. Checking the reading on my small barometer and thermometer, I trace my hand along the graph paper until I reach the point where humidity and temperature perfectly intersect. From here I take a third reading. A colour.

[SCREEN PAGE BREAK]

Silently waiting in the middle of my opened wooden box is a velvet handkerchief. I check my readings once again, retrace my fingers along the graph to ensure the certainty of my prediction. Once I am certain, and have readied myself a little for something I can anticipate but not describe, I pull the handkerchief away and see that the cactus, a precious gemstone that is perhaps the most valuable of all the artefacts I have recovered from these tribespeople, is precisely the colour I had expected: a rich burgundy-red.

Back in England, a sample this size of such an extraordinary material shall be highly desired by all major archaeological institutions. The British Geological Survey in particular ought to offer me a high price. As I take a moment to marvel at the wondrous stone, a fraction of which sits in the Elder's ring, the light diminishes in my tent as the night sky draws in. I turn quickly away from the cactus and check the thermometer. Sure enough, the temperature has dropped a significant fraction of a degree. I consult my chart again, aligning the new data, and produce a colour: deep purple with an orange iris. As though

 Copyright: CC-By Attribution 4.0 International

aligning itself to my new reading, the stone changes in less than a second and now appears dark purple with an outside band of orange which bleeds towards the centre. Thus I now feel utterly justified in concluding that the cactus's hitherto mysterious colour transformation is due solely to shifts in atmospheric conditions. Satisfied, I enfold the box back in on itself and pack it away with the rest of my equipment.

It appears to me only now that this presents another reason for my immediate departure: these tribespeople would be much displeased were they to find I had procured their religious artefact in the name of science and civilisation.

With all my instruments packed safely away, I set out up the winding track to the flatrock.

In my mind I begin to write my treatise on these remarkable indigenous folk. I begin to give the lectures which shall accompany the public display of the cactus. 'These remarkable indigenous folk,' I shall say, 'believe that this stone has deep spiritual significance. Yes,' I shall pause, 'during my time with the tribesfolk I was taken in as one of their own. They would talk to me, in their own inimitable rudimentary language, of the significance of the artefact you now see before you' (at which point I shall release the velvet drape to reveal the gemstone on a wonderful neoclassical stand). 'They would tell me tales, old stories delivered verbally (for they are yet to develop a formal writing system) through the generations, of a great monster. The monster, which the old tales name the carton, stalks the lowlands surrounding the village, killing and feeding on the great oxen which graze on the plains' (here I shall gesture to one of my many scientific drawings of these lumbering creatures).

[SCREEN PAGE BREAK]

It is raining heavily now, and my feet slip on the wet mud track. Above me I cannot see the peak beyond the dense foliage. Instead I keep my head down and continue my lecture. 'This monster, ladies and gentlemen, is said to appear as a friend, wearing as it does a wide smile at all times. The true horror of the creature, however, lies herein. The smile of the carton is meaningless. The monster wears the sign of human civility only in mockery, in arrogance. The carton is truly evil, a monster on two legs with a false smile which haunts both the plains and the dreams of the tribesfolk. Its sickly influence contorts one's nights into a realm of suffering, flooding one's peaceful thoughts with the intoxicating peril hidden behind every facade. The false smile. The horror. One cannot… is unable to… comprehend…' I look down upon the village, cloaked in darkness, and out over the lowlands. The wind howls and the rain beats down, but the twilight is silent. 'This is what the elders say, of course, and nothing more.'

 Copyright: CC-By Attribution 4.0 International

I turn my head to the peak once more. I have reached a turn in the track, and the rain falls down upon me heavier than ever in the clearing. I can see clearly now to the top. The last of the villagers are winding their way onto the flatrock and I know I must hurry. Slipping over wet rocks, I scramble up the track, now covered by dense foliage, now exposed to the biting wind and rain.

I continue to plan my lecture in my mind as I head up the track towards the peak. 'This cactus is of such significance to these people that they believe their gods communicate with them through its changing colours. They believe that because of this, the stone is able to predict the coming of the carton. When the Elder, an old man whom I knew very well, and who was exceedingly fond of me, I must say,' (laugh from the audience at my humorously narcissistic remark) 'holds up his ring on the great flatrock overlooking the plains, it is said that he shall determine whether the monster shall manifest during the coming hunting season… or perhaps whether it is among them already. At this point…' well, I hope to discover what these people do next when I reach the summit and observe the rites for myself.

'Obviously this is quite ludicrous. I have been able to objectively determine that the change in colour of the cactus is causally produced by the most mundane of all phenomena: the climate. Nothing more.' At this point I shall happily detail the research which has led me to this conclusion.

[SCREEN PAGE BREAK]

I near the peak now, and can hear floating notes on the wind, underneath the sound of rainfall. The short high notes of the small woodwinds dance around the deep roll of the long horns; the bruise is already playing their entrancing folk music.

Fog dancers wave their bodies in the rush of wind and music. The catastrophe of weather smashes onto the flatrock. The Elder holds the cactus ring out into the busy gloom with a rigid arm. The whole tribe appear to be performing the fog dance now, subtly shifting their bodies, trance-like. Booming winds and careful notes battle for dominance. Lightning then thunder begins to break forth from the heavens, the heavy sky at last tearing itself apart. The lecture hall in my mind is swept away and dashed across the lowlands. I have ascended the flatrock to find the village in a frenzy. Each eye is glazed as I try to shout to be heard.

I push my way through the throng, towards the Elder. Around him stands the bruise, in a ring, performing now with maniacal urgency. I shout, but the Elder's attention is transfixed by something high in the sky above. The rain streams across my face. My vision is blurred. I push through the crowd, desperate now, desperate for – for what? I do not know.

 Copyright: CC-By Attribution 4.0 International

‘Elder!’ I shout, in their language. At this, he starts. At least, I think it is at my call. No, it is something else. The cactus has changed. I look with disbelief. This colour – should it be this colour? I try to recall my charts, my findings, but come up short; my thoughts whip away with the wind. Should it be this colour? The Elder finally looks at me. The whole tribe looks at me. Should it be this colour? ‘The carton’ he shouts, stepping back. ‘The carton!’ It takes me a moment to translate. It takes me a little longer to translate the movements of the tribal people. Their gaze. They believe… They believe it is me. And the Elder holds his staff aloft with a fierce look in his eyes.

What happened next I have told not one person. In all my lectures, in all the years since the flatrock, I have never told a soul. I have never told anyone that when the old Elder held up his staff with a glint of madness in his eyes, the crowd forming solid ring all around him and me, the carton embodied, my mind grew cold with fear. I still cannot shake the sensation. I fell to my knees, crying, fearing what would come next, my heart began to beat faster than I ever thought possible. As the bruise’s music wrapped around my consciousness and the storm warped my vision, with the dark sky breaking into hellfire above me, the Elder urged me to take hold of his staff. I got to my feet and grabbed hold. At that very moment, a bolt of lightning plunged from the dark sky above and struck the staff. In a flash of blinding white light I was thrown backwards, pain searing through my body. All went quiet. To this day I don’t know how I survived it, without a single mark on my body. As I lay there on the flatrock, the storm quickly began to ease, my mind became filled with so many questions – how had the Elder known that the lightning would strike the staff? Surely it could not have been a coincidence. Through the haze of my thoughts, a singular idea was conceived: I was the carton embodied, and the lightning had purged me of the monster and saved the tribe. From *me*. There is no other explanation. There simply is no other explanation.

 Copyright: CC-By Attribution 4.0 International

Story 4: The Island and Elsewhere

We drew into the Island's only dock with the setting sun at our backs. The returning fishing vessels would soon be visible on the horizon. We had followed them the entire way, sitting just out beyond their view. It would have been impossible for them to spot us in our small rug; we had borrowed one of these traditional wooden fishing boats from the dock. We followed the black silhouettes of the large fishing vessels on the edge of the endless sea. Keeping pace was not a problem: rugs are crafted from the wood of the Island's most ancient trees and carved long and narrow, we were gliding through the waters with the speed and agility of a shark. One two-manned rug can skim over calm seas at unimaginable speeds.

'Are you satisfied Tane?' asked my good friend Maru. 'We have seen it many times now: the large boats do only one thing on the empty, endless sea' he said. 'They fish.'

The girl had been missing now for almost two lunar cycles. I had spoken to nigh every single person living in our rust, and in small island settlements such as our own we rely on each other as if part of one big family. We live in a typical rust: deep inland, almost at the centre of the Island in the only clearing of land, with the dense forest surrounding us all around. The trees give good shelter from the tropical rains, and our huts are huddled tightly together; everyone knows what's going on with everyone else in our close-knit community.

The only person, I soon learned, who had seen anything was an old woman, close to the end of her Island-life. I asked the woman several times if she was sure about what she had told me she had seen from the window of her hut. She insisted.

The old woman had seen the girl, Hana, with a *stranger*.

The world, as we knew it, consisted of only the Island and the endless sea. If you were to set out in a rug and keep a true, straight route across the wide ocean, you would eventually arrive back at the other end of the Island itself, having seen no other landmass, no other person, nothing else in the world but the sea and the Island. We few Islanders were humanity, and we had been blessed with this mound of land and vegetation to live out our long days.

And yet Hana was seen with a stranger.

[SCREEN PAGE BREAK]

I had always been an anomalous presence on the Island. Of all the villagers of the rust throughout our known history, I am the only one never to have been visited by the spirit of one of our ancient ancestors,

 Copyright: CC-By Attribution 4.0 International

who reside in the great fee: the vast, flat top of the Island's forest canopy, where vines and the thick foliage of all the trees are interwoven into one great expanse, stretching on towards the sea.

Because of this I am somewhat distrusted by the others who see me as *different*. So it naturally fell to me to investigate the anomalous, and I have been investigating rare or inexplicable occurrences for most of my life. But never, in all this time, have I ever had to look into something as rare or inexplicable as this.

Hana had been seen with a stranger, someone from *elsewhere*. With no elsewhere in the entire world, finding the missing girl seemed as impossible as her disappearance.

And so I met in secret with my only true friend, Maru. I told him what the old woman had seen and he believed me at once. Together, having become distrustful of the other Islanders, we arranged to follow the fishing fleet daily in one of the small two-manned rugs for as long as it took for us both to be satisfied that they were not dealing with some hidden group of people out there somewhere. Each morning we followed them out beyond the circle of trees protecting the rust, down towards the dock. From here we would watch them embark, wait until the boats were just visible in the distance, discretely board one of the small rugs, and set off in measured pursuit. For many days we continued this pattern, until we had followed the fleet around the entirety of the endless sea, sitting alone in our rug. Only then did Maru ask me if I was satisfied.

'Yes,' I said. 'Yes Maru, I am satisfied.'

'As am I,' he said, gazing out at the growing silhouettes in the distance. He turned to me. 'What will you do now, Tane? Where did this stranger come from, if he did not arrive from some unknown place in the endless sea?'

'I do not know,' I said. 'I shall attend the old ceremonies back in the rust once they begin, for it is a full moon tonight; perhaps nature shall finally open itself to me and I shall gain some valuable insight.'

[SCREEN PAGE BREAK]

The cold damp night blew in from the sea and the forest shivered. As I pushed forwards I came to a familiar opening in the clustered trees; I passed under ancient branches and into the protective warmth of the rust. An orange light held in the air, the fire in the centre of the clearing casting harsh, dancing shadows amongst the leaves of the surrounding trees. The ceremonies had begun.

The drummers controlled the rhythm of the night, which started with a slow but ominous beat. Already many were wearing their ceremonial headdresses, known as cakes, adorned with animal artefacts representing each domain of nature as we knew it; shells for the endless sea, feathers for the sky above the world, and fur for the creatures of the Island. The cake is traditionally worn in religious ceremonies

 Copyright: CC-By Attribution 4.0 International

on the island to celebrate our relationship with nature, and in wearing it we conjoin our souls with the flow of the natural world. Through the cake we could hear the Island *speak* to us.

I myself heard nothing, save the wind blowing through the empty shells of my own cake. I saw my fellow Islanders drunk on the hidden fruit of nature, dancing and rejoicing in the purity of life in all things. I thought of Hana and hoped she was alright.

The ceremony lasted long into the night, but I removed my cake early and set off to rest, just as the drums began to herald the real celebrations.

The night was late when I awoke in my resting place under a tall tree, away from the close-knit homes of the rust. With a heavy mind brought on by an unnatural waking I looked around. All was dim about me, save for a faint glow from faraway fires. Sleep came so easily on the Island, and dreams or any disruption of the peace were so rare that I found myself in a mild panic at having awoken before daylight. A little more alert, I sat up and moved my eyes rapidly, searching the thick undergrowth around me. Nothing. At some point I became aware of a slight coldness.

‘Hello, Tane’ came a voice. I could see, now, a man sitting cross-legged at my feet. I knew the voice and the man who carried it; sitting at my feet was Keola, a man so old that all the Islanders of his time were resting now with the ancestors in the great fee above. He had been cast out from the rust long ago; he had gone insane and the other Islanders soon grew tired of his nonsensical ravings. Now he lived nowhere, roaming the Island and cursing all those unfortunate enough to come across him.

[SCREEN PAGE BREAK]

‘My God is forever’ he said. ‘My God says nothing save what can be spoken, you see?’ He pointed to the old ruined cake resting on his head. Keola’s cake was unlike any other: adorned on either side with two great shells, curling like horns. He nodded and closed his eyes, ‘you see, you see. I see too’. He opened his eyes. ‘All is movement. All is resting. These things we know, you see?’ He held out his hands, imploring. ‘You will see. My God is wide, yes, but my God has eyes only to see what my God may know. My God’s being is bottomless. Yes, yes, my God sees all *within* but endlessly it stretches and my God… my God must turn away. All these things we know,’ he looked up, ‘but only those who feel the signs may bear it outward. Only those with ears to see and eyes to hear. Do you feel it?’ He pushed hard against my chest, then pointed to the canopy above. ‘Only those who *can* speak it, do.’

With that I fell into a deep sleep and dreamt of Hana and the stranger. When I awoke the next morning, I knew what to do.

 Copyright: CC-By Attribution 4.0 International

I climbed with the sun up to the canopy’s base. There I hesitated, for the great ancestral fee lay above me and I was afraid. With great effort, I pulled myself through the leaves and into another place.

Emerging onto the fee, one sees only vegetation – leaves and vines, all twisted and beautifully interwoven, with a thick layer of mist clinging to the top – endlessly stretching out into the horizon. It is said that were the fee an ocean, not even a fully-paced rug would ever find the end. A cool wind blew across the plane, and the leaves brushed against my arms. In the distance I suddenly heard what sounded like thunder, sustained and growing in intensity. Around me, the branches began to rustle and shake, and a horrific cracking filled the air. It appeared to me that the very substance of the fee was breaking itself apart and grouping into a mass before me. Then, amongst the movement, a shape began to form, looking more and more like a person with each new branch and leaf. I knew what was happening now. Many Islanders had seen it before me. I was being visited by an ancestor.

The ancestor sat a good three heads taller than myself, its head adorned with autumnal leaves arcing back, forming a natural imitation of a cake. ‘I knew I would see you this day, my ancestor’ I said, bowing my head slightly. ‘Old Keola seemed to be trying to tell me-‘

‘Keola is a fool,’ the ancestor spoke, his voice coming from somewhere I could not quite see. ‘And, Tane, I am not *your* ancestor. No, your ancestors do not reside with us.’ I opened my mouth to speak but the ancestor held up a hand and cut me off. ‘I have felt the changing of the winds, we all have, here. There are some of us who feel that the time is come. God is waking. Our work, begun many years ago, may finally be nearing an end. Which is why I come to you now, Tane.’

[SCREEN PAGE BREAK]

My mind began to tilt with the knowledge that my ancestors were not here, were not where I had looked up and spoken in futility all these long years. ‘Where else can they be?’ I said aloud. ‘The Island and the fee and the endless sea, do they not make up the world?’

The ancestor stared deep into my being. ‘You are correct, the island and the fee and the endless sea are all that comprise the world. Perhaps… perhaps this is not the entire tale, however. This Island, Tane, hides many secrets. There are those who wish these secrets to be revealed to one such as yourself, such that all may return to order. Change is coming, Tane, the great wave of time washes over us. It only remains to be seen who among us shall be standing once the tide rolls out again.’ The ancestor groaned a deep and harrowing groan. ‘You must go to the ancestral heart, Tane. All you need to know shall be revealed.’ With that, the ancestor let out a great sigh and collapsed into nothingness.

 Copyright: CC-By Attribution 4.0 International

My ascent to the heart of the forest, the ancestral heart, was beset at all times by vines and roots, forming from the undergrowth, pulling at me, back and away. The foliage thickened and spread before me. I could hear the distant sound of drums all around as I neared the centre of the Island. When I finally approached the heart-tree, the most ancient of trees, thick and tall, tremendous roots gripping the earth, I was very nearly defeated. As I neared the heart-tree, however, a chill wind blew from behind.

The old tree creaked and moaned in the wind. I looked up towards the top of the heart-tree, but I could see no further than a few thick branches. When I looked at the base of the tree, many thoughts came into being from the sight – the roots divided into large, hollow cracks before they penetrated the ground. Keola had said that his God's being was bottomless. It didn't occur to me at the time to wonder *which* God he could mean. The same God as the ancestors spoke of waking? The Island itself?

Without thinking, I impulsively strode into one of the dark cracks between the roots.

The track within wound down for what could have been a week, or perhaps an hour or a day. Eventually I arrived at a wide plateau, and stood on its precipice. I looked out over the edge, and saw a new world; a city sprawled out beneath the plateau for as far as I could see, falling away into expansive fields. Above this world hung a veil of mist, and standing there on the edge I could feel the cool wind from the sky. I knew I would find Hana here. The stranger had not come from outside of the Island, they had come from *within* it, and taken her with them.
