# Supplementary material for: The Role of Sleep in Learning New Meanings for Familiar Words through Stories
Source: J Cogn. 2023 Jun 15;6(1):27. doi: 10.5334/joc.282 (PMC10275344; doi:10.5334/joc.282)
Supplement: Table S1. — List of stimulus words and definitions of their meanings. [file joc-6-1-282-s2.pdf]

 Copyright: CC-By Attribution 4.0 International

**Table S1. List of stimulus words and definitions of their novel meanings.**

| Stimulus Word | Novel Meaning Definition |
|---|---|
| Story 1: *Pink Candy Dream* | |
| Hive | A new Chinese-made type of small car designed for inner-city living, with reduced boot space but extra storage in side pockets at the front of the car. |
| Vase | A colloquial term for a base used for criminal operations, they are chiefly used by big-city criminal gangs as places to meet in secret and carry out illegal dealings. |
| Path | The smallest surveillance device ever invented, it has a tiny camera through which it records and feeds back video, and is mobile and can be moved around by remote control. |
| Foam | A safe that is incorporated into a piece of furniture with a wooden panel concealing the key lock, and each is individually handcrafted so that no intruders are able to recognise the chief use of the furniture. |
| Story 2: *Prisons* | |
| Dawn | A biomedical implant fitted around a pacemaker to protect against electromagnetic interference, to which they are very susceptible, by acting as a barrier against electrical and magnetic signal. |
| Spy | The residual inner core left behind when a star dies, which are unique to each celestial body and can only be viewed through the world's most powerful telescopes. |
| Feast | A suit worn to protect against extremely high levels of harmful radiation, it covers the whole body with just a window to see through, but is particularly itchy and uncomfortable to wear. |

 Copyright: CC-By Attribution 4.0 International

| | |
|---|---|
| Pearl | A new medical device which is attached to the body and can take and record measurements from the blood without piercing the skin that can be transmitted to a receiver in hospital. |

Story 3: *Reflections upon a Tribe*

| | |
|---|---|
| Bruise | A type of traditional folk band which is made up of all male members, and when a player retires, their closest living relative is expected to take over their position which is considered a great honour. |
| Fog | A type of dance dating back centuries that is mainly performed by street performers, it involves elongating the body and swaying from side to side whilst keeping the head still. |
| Cactus | A unique and valuable type of precious stone that is often used in jewellery, it changes colour in a matter of seconds depending on the temperature and humidity. |
| Carton | A folkloric monster that walks on its two hind legs and has a fixed, mischievous smile, and is said to eat livestock. |

Story 4: *The Island and Elsewhere*

| | |
|---|---|
| Rug | A traditional type of wooden fishing boat used by communities on some Pacific islands, it requires two people to operate it and can move at a fast pace when the sea is calm. |
| Rust | The name for a small village in a clearing of land in the middle of the forest in which the houses are close together and the surrounding trees provide good shelter. |
| Fee | The name for the flat top of the forest canopy which is thick with different trees and plants interwoven; islanders believe it is the sacred realm of their ancestral spirits. |

 Copyright: CC-By Attribution 4.0 International

| | |
|---|---|
| Cake | A traditional tribal headdress decorated with feathers, shells and furs which is worn for religious ceremonies celebrating man’s relationship with nature, the land and the sea. |
