# Supplementary material for: The Role of Sleep in Learning New Meanings for Familiar Words through Stories
Source: J Cogn. 2023 Jun 15;6(1):27. doi: 10.5334/joc.282 (PMC10275344; doi:10.5334/joc.282)
Supplement: Table S2. — Stimulus words and paraphrased versions of their definitions. [file joc-6-1-282-s3.pdf]

 Copyright: CC-By Attribution 4.0 International

Table S2. Stimulus words and paraphrased versions of the definitions of their novel meanings used in the meaning-to-word test task.

Stimulus Word	Novel Meaning Definitions for Meaning-to-Word Matching Test
Story 1: *Pink Candy Dream*	
Hive	A new type of compact urban car made in China which has anterior side storage space but a small boot.
Vase	A slang name for a criminal gang's city headquarters where they meet to carry out illegal deals.
Path	A tiny remote-controlled piece of surveillance equipment that feeds back video from a minute camera.
Foam	A uniquely handcrafted item of furniture containing a safe with its lock hidden behind a wooden panel.
Story 2: *Prisons*	
Dawn	A medical device that is implanted around a pacemaker to shield it against interference from electromagnetic signals.
Spy	The unique inner remains left behind by dead stars which can only be viewed using the most powerful telescopes.
Feast	A protective suit which covers the whole body to guard against harmful radiation but is itchy and uncomfortable.
Pearl	A medical instrument that records and transmits readings from the blood without piercing the skin.
Story 3: *Reflections upon a Tribe*	
Bruise	A respected traditional folk band whose male members are replaced by their closest relative when they retire.

 Copyright: CC-By Attribution 4.0 International

Fog	An ancient dance in which the dancer, normally a street performer, lengthens and sways their body but holds their head still.
Cactus	A rare precious gemstone used in jewellery that changes colour instantly when the temperature and humidity change.
Carton	A beast of folklore that walks upright on two legs, has an evil smile and is believed to eat farm animals.

Story 4: *The Island and Elsewhere*

Rug	An old-fashioned two-man wooden fishing vessel that navigates calm seas quickly, it originates from some Pacific islands.
Rust	A small settlement in a forest glade with closely clustered houses and trees all around sheltering it.
Fee	The flat surface of intertwined plants and trees above the forest canopy that is home to islanders’ ancestral spirits.
Cake	A headdress traditionally worn by certain peoples in celebration of their relationship to nature, it is made of feathers, furs and shells.
