# Supplementary material for: The Role of Sleep in Learning New Meanings for Familiar Words through Stories
Source: J Cogn. 2023 Jun 15;6(1):27. doi: 10.5334/joc.282 (PMC10275344; doi:10.5334/joc.282)
Supplement: Table S3. — Target-probe word pairs used in the semantic relatedness judgement task. [file joc-6-1-282-s4.pdf]

 Copyright: CC-By Attribution 4.0 International

Table S3. Target-probe word pairs used in the semantic relatedness judgement task.

Target Word	Item Type	Related Probe	Unrelated Probe
Hive	Trained	Honey	Bicycle
Vase	Trained	Flower	Fox
Path	Trained	Trail	Pillow
Foam	Trained	Soap	Belt
Dawn	Trained	Dusk	Basket
Spy	Trained	Mission	Fungus
Feast	Trained	Banquet	Skull
Pearl	Trained	Jewel	Battery
Bruise	Trained	Injury	Address
Fog	Trained	Cloud	Blade
Cactus	Trained	Plant	Doll
Carton	Trained	Juice	Alarm
Rug	Trained	Mat	Rocket
Rust	Trained	Iron	Comedy
Fee	Trained	Payment	Cliff
Cake	Trained	Dough	Alien
Untrained control items included in Experiment 2 only:			
Shield	Untrained control	Sword	Baker
Barber	Untrained control	Razor	Basil

 Copyright: CC-By Attribution 4.0 International

Shoe	Untrained control	Sock	Goose
Wool	Untrained control	Cotton	Eagle
Frost	Untrained control	Winter	Golf
Beef	Untrained control	Cow	Blouse
Grain	Untrained control	Rice	Kiss
Torch	Untrained control	Bulb	Elbow
