# Supplementary material for: The Role of Sleep in Learning New Meanings for Familiar Words through Stories
Source: J Cogn. 2023 Jun 15;6(1):27. doi: 10.5334/joc.282 (PMC10275344; doi:10.5334/joc.282)
Supplement: Table S4. — Properties of the probe words in the semantic relatedness task in Experiment 1. [file joc-6-1-282-s5.pdf]

 Copyright: CC-By Attribution 4.0 International

Table S4. Descriptive statistics for the lexical and semantic properties of the probe words used in the semantic relatedness judgement task in Experiment 1.

The means for each measure are displayed in the table, with standard deviations given in parentheses. The word frequency data reported are SUBTLEX-UK word frequencies in occurrences per million and log-transformations of the raw word frequencies (log10[raw frequency+1]) (van Heuven et al., 2014). Word sense data are from the WordNet (Fellbaum, 1998) and Wordsmyth (Parks et al., 1998) dictionaries. The number of semantic associates counts come from Nelson et al. (2004). The target-probe semantic relatedness values are Latent Semantic Analysis (LSA) estimates (Landauer et al., 1998).

	Related Probes	Unrelated Probes
Example	*dawn-dusk*	*dawn-basket*
Number of Letters	5.19 (1.17)	5.44 (1.15)
Frequency (per mil.)	26.28 (19.38)	17.59 (12.05)
Frequency (log-transf.)	3.58 (0.41)	3.45 (0.32)
WordNet Senses	5.56 (3.44)	5.50 (4.66)
Wordsmyth Senses	6.19 (4.05)	4.88 (2.75)
Number of Semantic Associates	14.69 (5.26)	14.27 (3.69)[1]
Target-Probe Relatedness	0.44 (0.18)	0.06 (0.07)

[1] There was no data for one item (*alien*, which was the semantically unrelated probe for *cake*).

 Copyright: CC-By Attribution 4.0 International

References

Fellbaum, C. (1998). *WordNet: An Electronic Lexical Database*. MIT Press.

Landauer, T. K., Foltz, P. W., & Laham, D. (1998). An introduction to latent semantic analysis. *Discourse Processes*, *25*(2–3), 259–284. https://doi.org/10.1080/01638539809545028

Nelson, D. L., McEvoy, C. L., & Schreiber, T. A. (2004). The University of South Florida free association, rhyme, and word fragment norms. *Behavior Research Methods, Instruments, & Computers*, *36*(3), 402–407. https://doi.org/10.3758/BF03195588

Parks, R., Ray, J., & Bland, S. (1998). *Wordsmyth English Dictionary-Thesaurus [Electronic version]*. University of Chicago. https://www.wordsmyth.net

van Heuven, W. J. B., Mandera, P., Keuleers, E., & Brysbaert, M. (2014). SUBTLEX-UK: A new and improved word frequency database for British English. *The Quarterly Journal of Experimental Psychology*, *67*(6), 1176–1190. https://doi.org/10.1080/17470218.2013.850521
