# Supplementary material for: The Role of Sleep in Learning New Meanings for Familiar Words through Stories
Source: J Cogn. 2023 Jun 15;6(1):27. doi: 10.5334/joc.282 (PMC10275344; doi:10.5334/joc.282)
Supplement: Supplementary Results. — Alternative Simple Effects Analyses. [file joc-6-1-282-s7.pdf]

 Copyright: CC-By Attribution 4.0 International

Supplementary Results: Alternative Simple Effects Analyses

These were the preregistered simple effects analyses for Experiment 2 that examined the effect of training within the two groups.

Cued Recall of Meanings

The planned simple effects follow-up analysis (see Figure I) showed that for the AM test group cued recall accuracy was significantly higher for the 12-hour delay (items that were trained in the second training session, in the evening just prior to sleep; 27.0%) than the 24-hour delay (14.8%) [$\chi^2(1) = 11.45$, $p < .001$; $\alpha = .025$]. However, this was not the case for the PM test group who showed no significant difference in accuracy between the 24-hour delay (16.5%) and the 12-hour delay (17.4%) [$\chi^2(1) = 0.10$, $p = .750$; $\alpha = .025$].

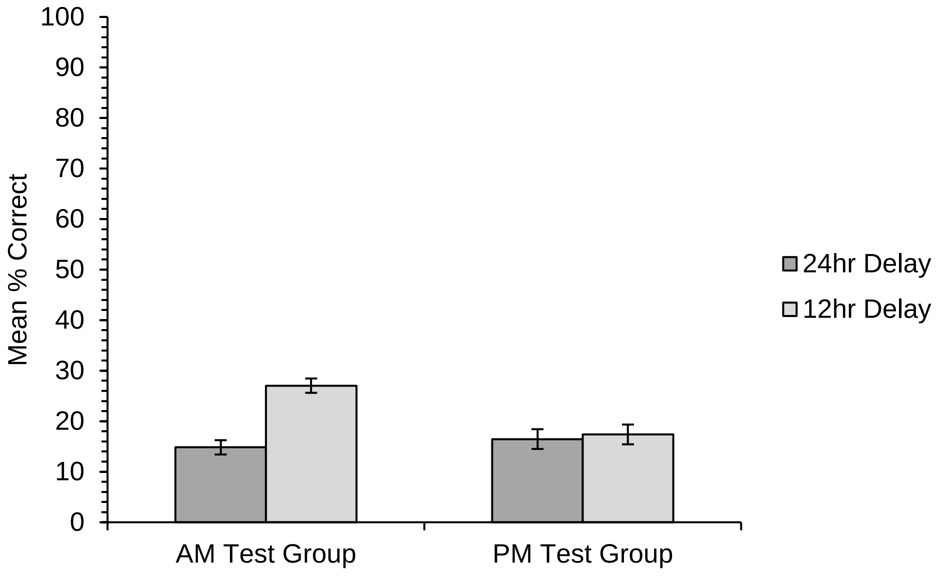

Figure I. *Experiment 2. Mean percentage of correct responses on the cued recall test (meanings correctly recalled for the appropriate word) by participants in the AM test group and PM test group for new word meanings trained either 12 hours or 24 hours prior to test. Error bars show standard errors for the means, adjusted for the within-participants factor of training condition (Cousineau, 2005).*

 Copyright: CC-By Attribution 4.0 International

Multiple-choice meaning-to-word matching

The planned simple effects follow-up analysis (see Figure II) showed that for the AM test group accuracy was again significantly higher for the 12-hour delay (items trained in the second training session, in the evening just prior to sleep; 56.7%) than the 24-hour delay (46.8%) [$\chi^2(1) = 5.29$, $p = .021$; $\alpha = .025$]. For the PM test group there was no significant difference in accuracy between the 24-hour delay (52.7%) and the 12-hour delay (53.7%) [$\chi^2(1) = 0.00$, $p = .964$; $\alpha = .025$].

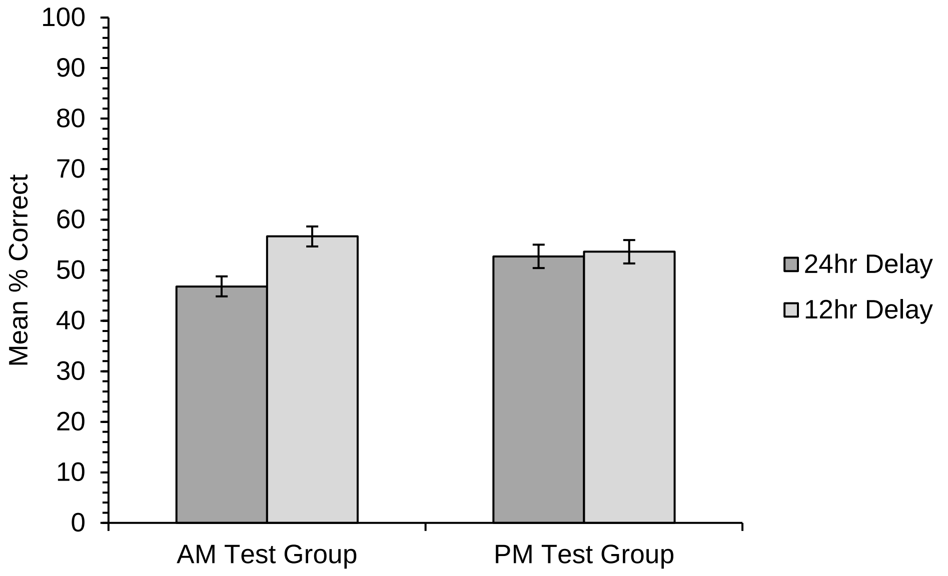

Figure II. *Experiment 2. Mean percentage of correct responses on the multiple-choice meaning-to-word matching test (words correctly paired with the appropriate definition) by participants in the AM test group and PM test group for new word meanings trained either 12 hours or 24 hours prior to test. Error bars show standard errors for the subject means, adjusted for the within-participants factor of training condition (Cousineau, 2005).*

Semantic relatedness judgement

The planned follow-up simple effects analyses showed that for the AM test group there was no significant effect for untrained compared with 12-hour delay [$\chi^2(1) = 0.0001$, $p = .991$],

 Copyright: CC-By Attribution 4.0 International

untrained compared with 24-hour delay [$\chi^2(1) = 0.07$, $p = .791$], nor the 12-hour compared with the 24-hour delay [$\chi^2(1) = 0.45$, $p = .504$]. For the PM test group the comparison between the untrained and 12-hour delay was significant at the non-corrected level of $\alpha = .05$ [$\chi^2(1) = 3.90$, $p = .048$], but not at the Bonferroni-corrected level of $\alpha = .008$. The comparison between untrained and 24-hour delay [$\chi^2(1) = 1.25$, $p = .264$], and the comparison between the 12-hour and 24-hour delays was also non-significant [$\chi^2(1) = 1.41$, $p = .235$].

Semantic relatedness judgement task exploratory analysis

The simple effects analyses showed that for the AM test group there was no significant effect for untrained compared with 12-hour delay [$\chi^2(1) = 0.02$, $p = .896$], untrained compared with 24-hour delay [$\chi^2(1) = 0.004$, $p = .953$], nor the 12-hour compared with the 24-hour delay [$\chi^2(1) = 0.04$, $p = .844$]. For the PM test group the comparison between the untrained and 12-hour delay was non-significant [$\chi^2(1) = 0.59$, $p = .442$], as was the comparison between untrained and 24-hour delay [$\chi^2(1) = 1.37$, $p = .241$], and the comparison between the 12-hour and 24-hour delays [$\chi^2(1) = 0.05$, $p = .823$]. (The p-values for the simple effects analyses were compared against a Bonferroni-corrected α of .008.)
