# Supplementary material for: The Role of Sleep in Learning New Meanings for Familiar Words through Stories
Source: J Cogn. 2023 Jun 15;6(1):27. doi: 10.5334/joc.282 (PMC10275344; doi:10.5334/joc.282)
Supplement: Supplementary Results. — Exploratory Analyses. [file joc-6-1-282-s8.pdf]

 Copyright: CC-By Attribution 4.0 International

Supplementary Results: Exploratory Analyses

These exploratory analyses of data from the semantic relatedness judgement task were carried out with a subset of trained items for which participants had correctly recalled the item in the cued recall test. These analyses were carried out in the same way as the main analyses.

Experiment 1 semantic relatedness judgement task exploratory analysis: Items correctly recalled

The models for both the RT and accuracy analyses used the maximal random effects structure simplified by removing the correlations between the by-participant and by-item random slopes and random intercepts (Barr et al., 2013). The RT analysis was carried out with inverse-transformed RTs.

The mean percentage error rates for the subset of data for only trained items that had been correctly recalled by participants in the subsequent cued recall test can be seen in Figure I. The main effect of group was non-significant [$\chi^2(1) = 0.01$, $p = .905$], as was the main effect of training [$\chi^2(1) = 0.24$, $p = .627$], and the interaction between group and training [$\chi^2(1) = 1.04$, $p = .309$]. The main effect of block and all of the other interactions were also non-significant (all $p > .05$).

 Copyright: CC-By Attribution 4.0 International

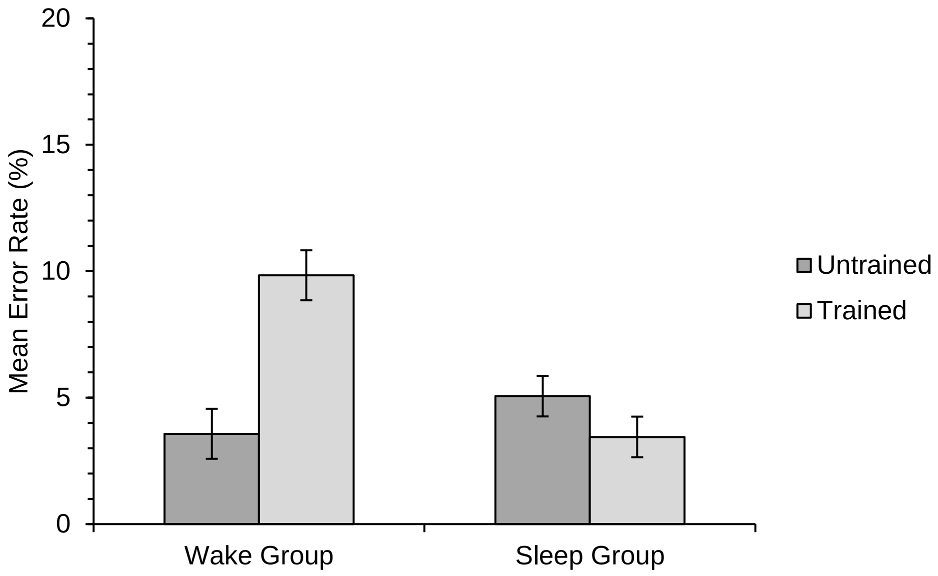

Figure I. *Experiment 1. Mean percentage error rate on the semantic relatedness judgement task for participants in each of the two groups for untrained and trained items only for the subset of trained items that participants correctly recalled in the cued recall test. The data shown are related trials only (trials in which the target and probe were semantically related). Error bars show standard errors for the means, corrected for the within-participants factor of training condition (Cousineau, 2005).*

The subset of RT data for only trained items that had been correctly recalled by participants in the subsequent cued recall test can be seen in Figure II. There was a marginally non-significant main effect of group [$\chi^2(1) = 3.22$, $p = .073$], and no significant main effect of training [$\chi^2(1) = 0.82$, $p = .366$] or training by sleep group interaction [$\chi^2(1) = 0.01$, $p = .903$]. The main effect of block and all of the other interactions were also non-significant (all $p > .05$).

 Copyright: CC-By Attribution 4.0 International

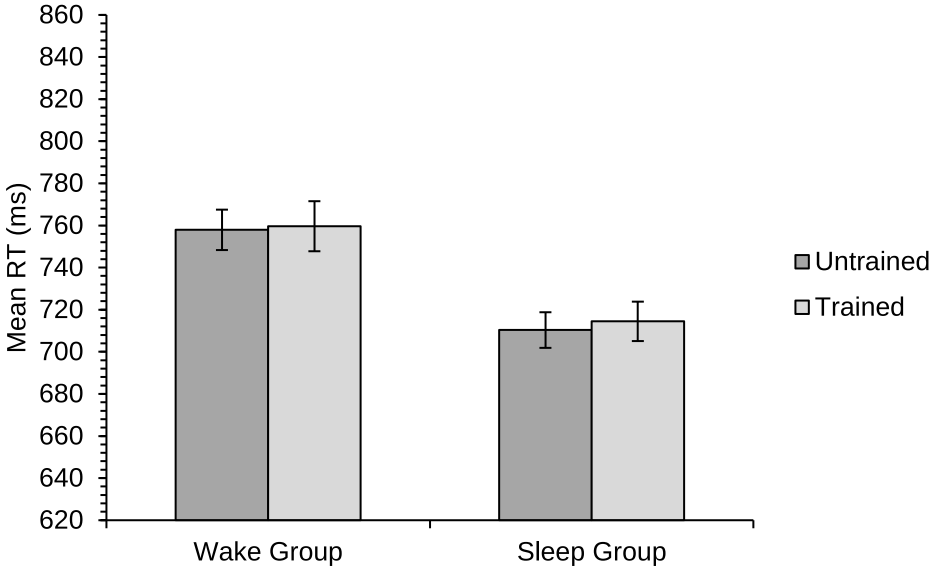

Figure II. *Experiment 1. Mean reaction time on the semantic relatedness judgement task for participants in each of the two groups for untrained and trained items only for the subset of trained items that participants correctly recalled in the cued recall test. The data shown are for correct related trials only (trials to which the participants correctly responded 'yes' that the target and probe were semantically related). Error bars show standard errors for the means, corrected for the within-participants factor of training condition (Cousineau, 2005).*

Experiment 2 semantic relatedness judgement task exploratory analysis: Items correctly recalled

The models for both the RT and accuracy analyses used the maximal random effects structure, the random effects structure for the accuracy analysis was simplified by removing the correlations between the by-participant and by-item random slopes and random intercepts (Barr et al., 2013). The RT analysis was carried out with inverse-transformed RTs.

The mean percentage error rates for the subset of data for only trained items that had been correctly recalled by participants in the subsequent cued recall test can be seen in Figure III. The main effect of group was non-significant [$\chi^2(1) = 0.38$, $p = .537$], as was the main effect of

 Copyright: CC-By Attribution 4.0 International

training [$\chi^2(2) = 0.65$, $p = .721$], and the interaction between group and training [$\chi^2(2) = 1.95$, $p = .377$].

The planned follow-up analyses of the three two-way interactions between group and pairs of levels of training condition showed no significant interaction between group and training for the untrained and 12-hour delay conditions [$\chi^2(1) = 0.17$, $p = .684$], the untrained and 24-hour delay conditions [$\chi^2(1) = 2.08$, $p = .150$], nor the 12-hour and 24-hour delay conditions [$\chi^2(1) = 0.68$, $p = .409$; $\alpha = .017$]. The simple effects analyses showed no effect of group for the untrained condition [$\chi^2(1) = 0.63$, $p = .426$], the 12-hour delay condition [$\chi^2(1) = 0.06$, $p = .801$], nor the 24-hour delay condition [$\chi^2(1) = 0.70$, $p = .402$; $\alpha = .017$].

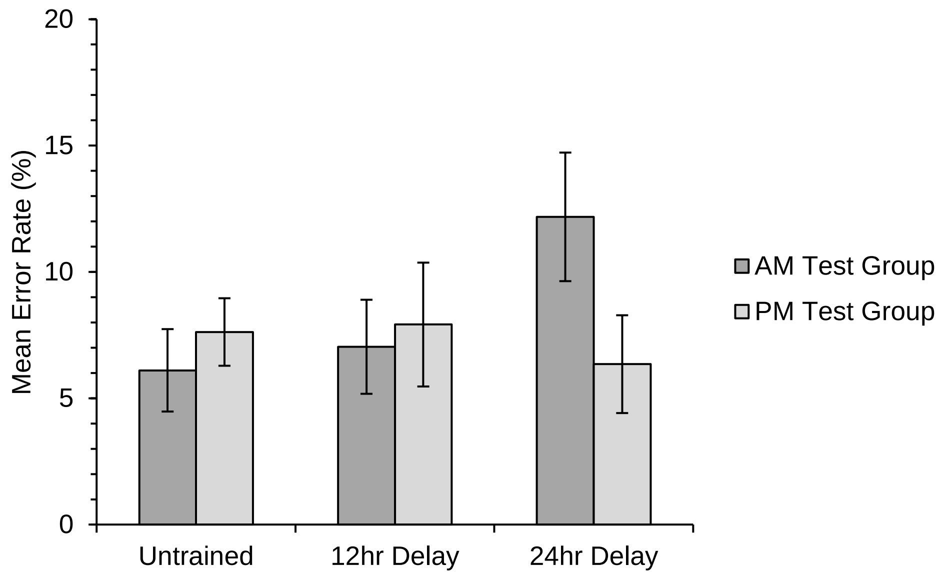

Figure II. *Experiment 1. Mean percentage error rate on the semantic relatedness judgement task for participants in each of the two groups for untrained and trained items only for the subset of trained items that participants correctly recalled in the cued recall test. The data shown are related trials only (trials in which the target and probe were semantically related). Error bars show standard errors for the means, corrected for the within-participants factor of training condition (Cousineau, 2005).*

 Copyright: CC-By Attribution 4.0 International

The subset of RT data for only trained items that had been correctly recalled by participants in the subsequent cued recall test can be seen in Figure IV. There was a non-significant main effect of group [$\chi^2(1) = 2.89$, $p = .089$], and no significant main effect of training [$\chi^2(2) = 0.66$, $p = .720$] or training by group interaction [$\chi^2(2) = 1.04$, $p = .595$].

The planned follow-up analyses of the three two-way interactions between group and pairs of levels of training condition showed no significant interaction between group and training for the untrained and 12-hour delay conditions [$\chi^2(1) = 0.54$, $p = .464$], the untrained and 24-hour delay conditions [$\chi^2(1) = 0.89$, $p = .344$], nor the 12-hour and 24-hour delay conditions [$\chi^2(1) = 0.02$, $p = .897$; $\alpha = .017$]. The simple effects analyses showed that the PM test group were faster than the AM test group in the untrained condition [$\chi^2(1) = 7.14$, $p = .008$]. There was no effect of group for the 12-hour delay condition [$\chi^2(1) = 1.21$, $p = .270$], nor the 24-hour delay condition [$\chi^2(1) = 0.68$, $p = .410$; $\alpha = .017$].

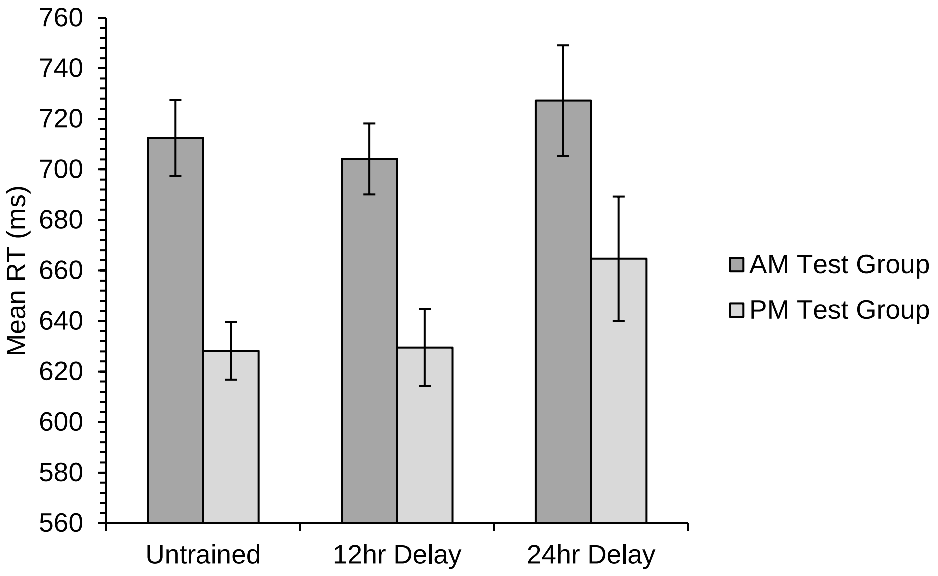

Figure IV. *Experiment 1. Mean reaction time on the semantic relatedness judgement task for participants in each of the two groups for untrained and trained items only for the subset of trained items that participants correctly recalled in the cued recall test. The data shown are for correct related trials only (trials to which the participants correctly responded 'yes' that the target and probe were semantically*

 Copyright: CC-By Attribution 4.0 International

related). Error bars show standard errors for the means, corrected for the within-participants factor of training condition (Cousineau, 2005).
